# Risk perception and behavioral change during epidemics: Comparing models of individual and collective learning

**DOI:** 10.1371/journal.pone.0226483

**Published:** 2020-01-06

**Authors:** Shaheen A. Abdulkareem, Ellen-Wien Augustijn, Tatiana Filatova, Katarzyna Musial, Yaseen T. Mustafa

**Affiliations:** 1 Center of Studies of Technology and Sustainability Development (CSTM), Faculty of Behavioral, Management, and Social sciences (BMS), University of Twente, Enschede, The Netherlands; 2 Department of Computer Science, College of Science, University of Duhok (UoD), Kurdistan region, Iraq; 3 Department of Geo-Information Processing (GIP), Faculty of Geo-Information Science and Earth Observation (ITC), University of Twente, Enschede, The Netherlands; 4 School of Information, Systems and Modeling, Faculty of Engineering and Information Technology, University of Technology Sydney (UTS), Sydney, Australia; 5 Advanced Analytics Institute, School of Software, Faculty of Engineering and IT, University of Technology Sydney (UTS), Sydney, Australia; 6 Faculty of Science, University of Zakho (UoZ), Kurdistan region, Iraq; INSERM, FRANCE

## Abstract

Modern societies are exposed to a myriad of risks ranging from disease to natural hazards and technological disruptions. Exploring how the awareness of risk spreads and how it triggers a diffusion of coping strategies is prominent in the research agenda of various domains. It requires a deep understanding of how individuals perceive risks and communicate about the effectiveness of protective measures, highlighting learning and social interaction as the core mechanisms driving such processes. Methodological approaches that range from purely physics-based diffusion models to data-driven environmental methods rely on agent-based modeling to accommodate context-dependent learning and social interactions in a diffusion process. Mixing agent-based modeling with data-driven machine learning has become popularity. However, little attention has been paid to the role of intelligent learning in risk appraisal and protective decisions, whether used in an individual or a collective process. The differences between collective learning and individual learning have not been sufficiently explored in diffusion modeling in general and in agent-based models of socio-environmental systems in particular. To address this research gap, we explored the implications of intelligent learning on the gradient from individual to collective learning, using an agent-based model enhanced by machine learning. Our simulation experiments showed that individual intelligent judgement about risks and the selection of coping strategies by groups with majority votes were outperformed by leader-based groups and even individuals deciding alone. Social interactions appeared essential for both individual learning and group learning. The choice of how to represent social learning in an agent-based model could be driven by existing cultural and social norms prevalent in a modeled society.

## 1 Introduction

When facing risks, people go through a complex process of collecting information, deciding what to do, and communicating with others about the effectiveness of their actions. Social influence may interfere with personal experiences, making peer groups and group interactions important factors. This is especially important in understanding disease diffusion and the emergence of epidemics, as these phenomena annually take thousands of lives worldwide [1]. Hence, good responsive and preventive strategies at both the individual and government levels are vital for saving lives. A choice of strategy depends on behavioral aspects, complex interactions among people [2], and the information available about a disease [3]. Perceiving the risk of an infectious disease may trigger behavioral change, as during the 2003 SARS epidemic [4]. Gathering information and experience through multiple sources is essential for increasing disease risk awareness about the disease and taking protective measures [5]. To help prevent epidemics, we need advanced tools that identify the factors that help spread of information about life-threatening diseases and that change individual behavior to curbs the diffusion of disease.

Various scientific approaches have been developed to tackle this challenge. Network science is prominent in studying how epidemics propagate and how different awareness mechanisms can help to prevent the outbreak of disease. Some researchers propose a framework with different mechanisms for spreading awareness about a disease as an additional contagion process [6]. Others model populations as multiplex networks where the disease spreads over one layer and awareness spreads over another [7]. The influence of the perception of risk on the probability of infection also has been studied [8]. Several recent studies have shown how information spreads in complex networks [9,10]. However, a different approach is needed to account for individual heterogeneity (such as income and education levels), the richness of the information on social and spatial distance or media influence. Here, a combination of modeling with data-driven machine learning becomes particularly attractive. Simulation tools are commonly used to assess the effects of policy impacts in the health domain [3,11,12]. Among the models for policy-making, agent-based modeling (ABM) is recommended as the most promising modeling approach [13]. ABM studies the dynamics of complex systems by simulating an array of heterogeneous individuals that make decisions, interact with each other, and learn from their experiences and the environment. The method is widely used to analyze epidemics [14–17]. Its advantage is in analyzing the factors that influence the spread of infectious diseases and the actions of individual actors [18]. As a bottom-up method, ABM integrates micro-macro relationships while accommodating agents’ heterogeneity and their adaptive behavior. It ensures that the interaction between the spatial environment and the behavior agents can integrate a variety of data inputs including aggregated, disaggregated and qualitative data [19–22].

Two processes are essential in representing agents’ health behavior and disease dynamics, the evolution of risk perception, and selection of a coping strategy. Hence, the core of a disease ABM lies in defining the learning methods that steer these two processes. Sensing of information (global, from the environment, and social, i.e., from other agents), exchanging information (i.e., interactions between agents), and processing of information (i.e., decision making) are critical. Machine learning (ML) techniques can support these three elements and offer a more realistic way to adjust agents’ behavior in ABM [23–26]. As more data become available in the analysis of the spread of disease, supporting ABM with data-driven approaches becomes a prominent research direction. ML has the potential to enhance ABM performance, especially when the number of agents is large (e.g., pandemics) and the decision-making process is complex (e.g., depending on both past experience and new information from the environment and peers).

ML approaches in ABM can provide agents with the ability to learn by adapting their decision-making process in line with new information. People make decisions both as individuals and as members of a group who imitate the decisions taken by the group or its leader [27]. Information about social networks is becoming increasingly available, e.g., through social media analysis. It may reveal collective behavior in various domains, including health [28]. For example, people are not entirely rational and imitate others in their views about vaccines [29]. Many ABMs rely solely on the decisions of individuals, paying little attention to group behavior [30]. Yet, mirroring emotions, beliefs, and intentions in an ABM with the collective decision making of crowds affects social contagion in ABMs [31].

Agents–individuals and groups–may learn in isolation or through interactions with others, such as their neighbors [32]. In isolated learning, agents learn independently, requiring no interaction with other agents. In interactive learning, several agents are engaged in sensing and processing information and communicating and cooperating to learn effectively. Interactive learning can be done in multiple ways, i.e., based on different social learning strategies [33]. Agents might be represented as members of local groups, learning together and mimicking behavior from other group members (i.e., collective learning) [34]. Yet, the impact of different types of interactive learning in groups compared to learning by an individual is an under-explored domain in the development of ABMs of socio-environmental systems.

This article examines the influence of individual vs group learning on a decision-making process in ABMs enhanced with ML. To illustrate the implications of individual and collective intelligence in ABMs, we used a spatially explicit disease model of cholera diffusion [35] as a case study. Bayesian Networks (BNs) steer agents’ behavior when judging on risk perception (RP) and coping appraisal (CA). We quantitatively tested the influence of agents’ ability to learn–individually or in a group–on the dynamics of disease. The main goal is, therefore, methodological: to introduce ML into a spatial ABM with a focus on comparing individual learning to collective learning. The added value of the analysis of alternative implementations of learning in ABMs goes beyond the domain of disease modeling. It illustrates the effects of individuals learning and collective learning on the field of ABMs of socio-environmental systems as a whole. Therefore, our main objectives are to (1) simulate the learning processes of agents on a gradient of learning from individual to collective, and (2) understand how these learning processes reveal the dynamics of social interactions and their emergent features during an epidemic. To address these objectives, the article aims to answer the following research questions: (*RQ1*) What is the impact of social interactions on the perceptions and decisions of intelligent individuals facing a risk? (*RQ2*) How do different implementations of group learning–deciding by majority voting vs by leaders–impact the diffusion process? (*RQ3*) What are the implications of implementing collective learning for risk assessment combined with individual coping strategies? By answering these methodological questions for our case study, we reveal whether individuals perform better than groups at perceiving risks and at coping during epidemics.

## 2 Methods

To explore the implications of intelligent learning on the gradient from individual to collective, we advance the existing cholera ABM (CABM) originally developed to study cholera diffusion [35]. In CABM, MLs steer agents’ behavior [23,35,36], helping them to adjust risk perception and coping during an epidemic outbreak. For this study, we ran eight ABMs to test various combinations of individual and group learning, using different information sources–with or without interactions among agents–as factors in the BNs. We investigate the extent to which the epidemic spreads, depending on these different learning approaches regarding risk perception and coping decisions. S1 Appendix provides a technical description of the model and the MLs. Below we briefly outline the processes in CABM essential to understand the performed simulation experiments.

### 2.1 Case study: Cholera diffusion ABM

Nowadays, 69 countries worldwide are labeled as cholera-endemic, with 2.8 million cases each year leading to 91,000 deaths [37]. People in urban slums and refugee camps are at high risk of cholera because of limited or no access to clean water and adequate sanitation. CABM is an empirically and theoretically grounded model developed to study the 2005 cholera outbreak in Kumasi, Ghana [35]. The open-source code for the model code is available online.

**Fig 1:**
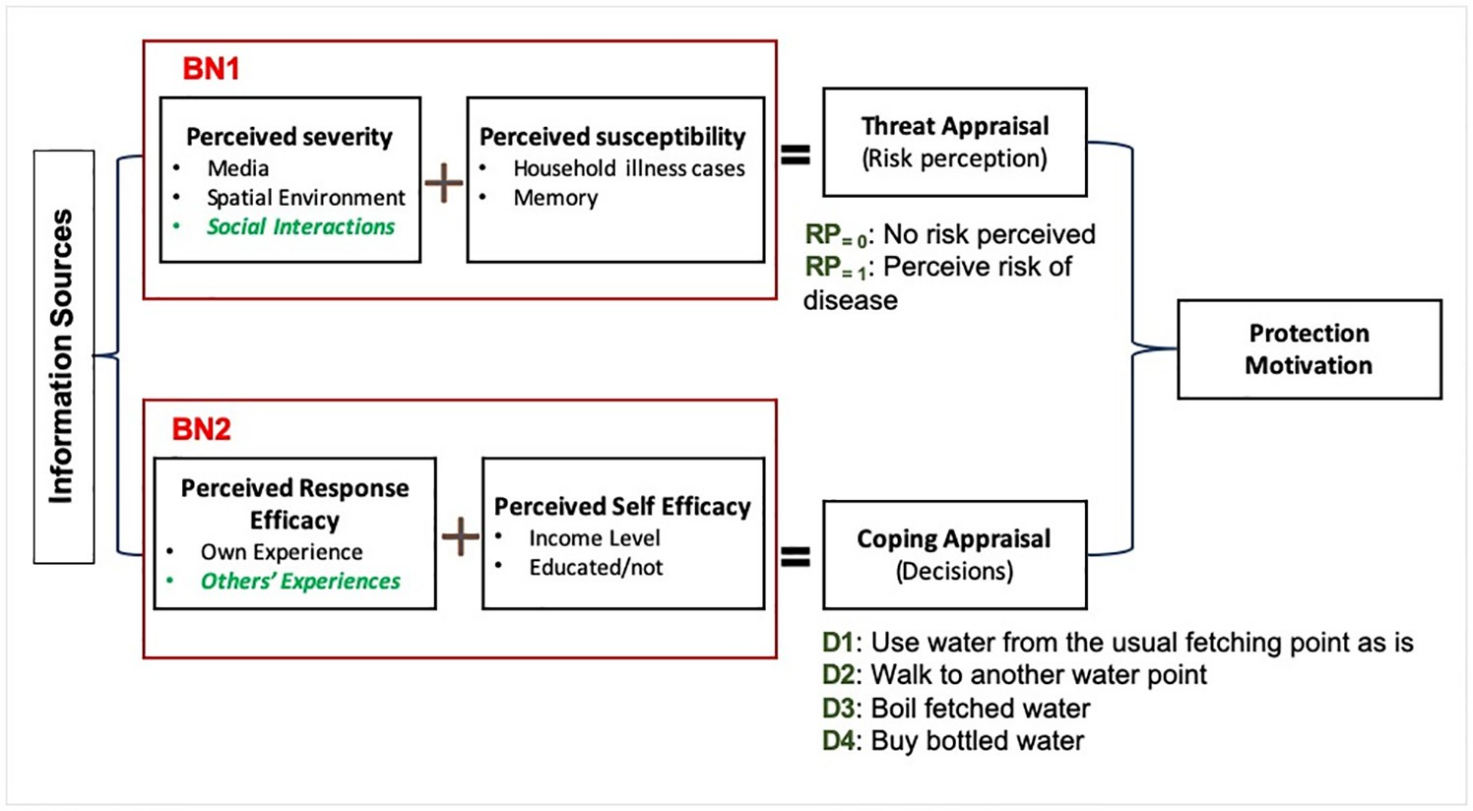
Framework of Bayesian Networks representing threat and coping appraisal Protection Motivation Theory for health behavior (adapted from Rogers, 1975).

CABM is grounded in the Protection Motivation Theory (PMT) in psychology [23,38]. The empirically-driven BNs model a two-stage decision process of people facing a disease risk: learning to update risk perceptions (threat appraisal, BN1 in Fig 1) and making decisions about how to adapt their behavior during the epidemic (coping appraisal, BN2 in Fig 1).

According to PMT, threat appraisal depends on individual perceptions of the severity of the disease (evaluating the state of the environment and observing what happens to others) and one’s own susceptibility. The coping appraisal is driven by the perceived response efficacy (the belief that the recommended behavior will protect) and one’s own self-efficacy (the ability to perform the recommended behavior).

**Fig 2:**
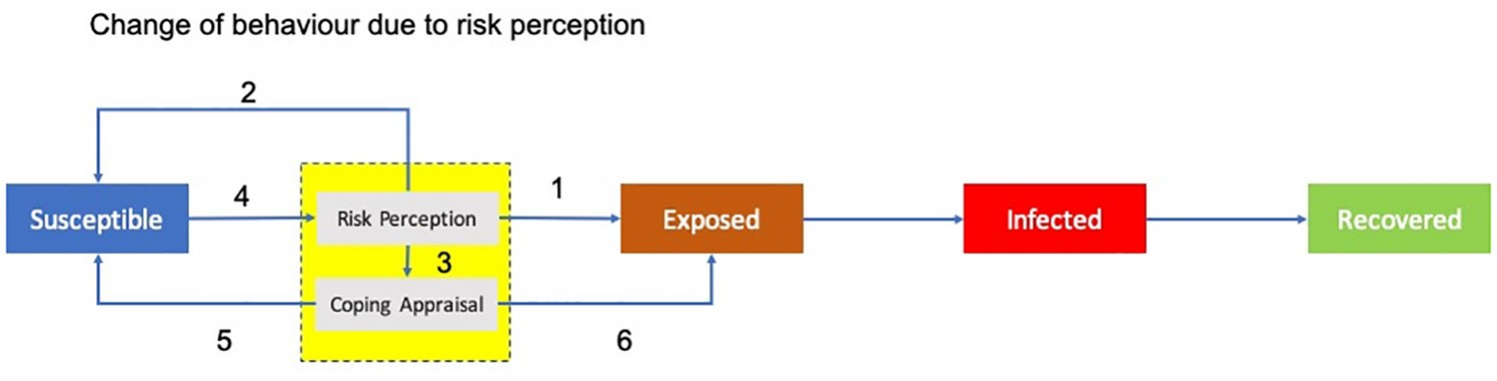
Adjustment of the SEIR model in CABM.

CABM simulates individuals who are spatially located in a city. These agents differ by income and education level. Individual agents form households and neighborhood groups and are susceptible to cholera at the beginning of the simulation. CABM implements an adjusted SEIR model [39] as explained in Fig 2 below.

Instead of going directly from Susceptible to Exposure, we introduced an awareness component in which agents can assess their risk. Options included: no risk perception in which the agent will be exposed (arrow 1, Fig 2); no risk perception yet no exposure (arrow 2, Fig 2); and risk perception leading agents to the coping phase (arrow 3, Fig 2). Exposure to cholera takes place through the use of unsafe river water. Agents can influence their exposure by selecting alternative water sources. These alternative water sources can either reduce their exposure to zero (arrow 5, Fig 2) or have no effect on their infection risk (arrow 4, Fig 2). Their actions are contingent on income and education levels, as well as on the information that they retrieve from their own experience, information received from others, or observations of the environment. It is not possible to judge by sight whether surface water is infected with cholera, but the agents use other types of visual pollution, e.g., floating garbage, as a proxy. When household agents find the visual pollution level too high, they may decide on an alternative. Household agents with high incomes do not take a risk and will buy safe water.

In CABM, the risk perception was updated using BN1, which depends on the agent memory (Me), the visual pollution at the water fetching point (VP), and the evidence of the severity of the epidemic based on communication from the media (M) and potentially with neighbor households (CNH). Media broadcast news about cholera starting on day 21 onward (see: Ghana News Archive). During a simulation, household agents may also interact with their neighbors zero to seven times a day (applied randomly) [40]. When interactive learning was activated, social interactions among household agents helped to share information on cholera cases that occurred in their communities and on the effectiveness of coping decisions. If risk perception was positive (BN1 returns a value above 0.5), household agents activate BN2 to decide which action (D1 –D4, Fig 1) to take given their income (I) and education (E) level, the experience of their own household with cholera (OE), and possibly their neighbors’ experiences with cholera (NE) [22]. S1 Appendix provides further details on how the BNs are implemented, together with tables of the parameters. Sensitivity analysis of the aggregated model dynamics on the BNs inputs and training alternatives can be found in [23, 36].

### 2.2 From individual to collective intelligence: Defining the gradient of learning strategies

**Fig 3:**
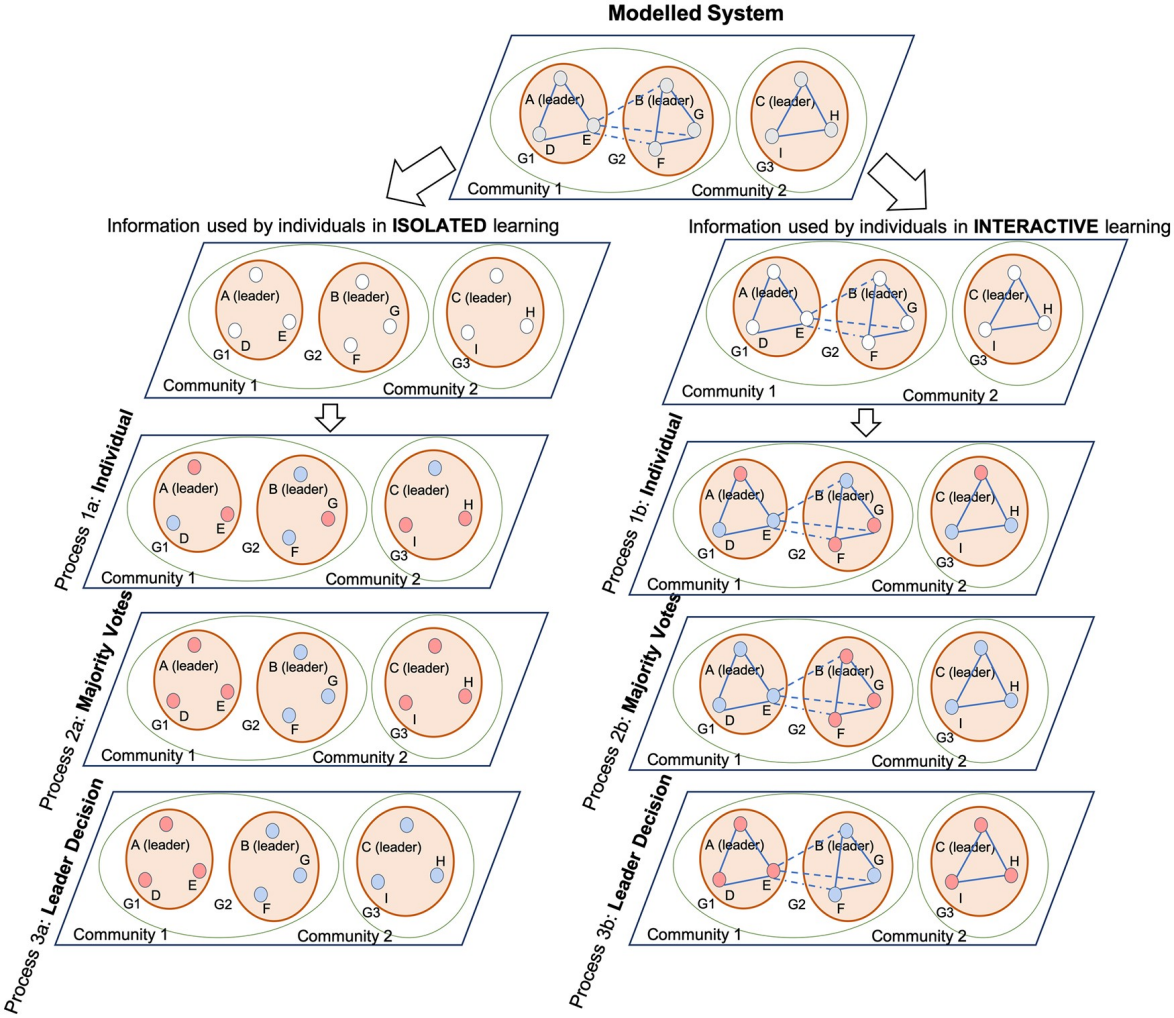
Agents’ learning types in cholera ABM. G1, G2, and G3 indicate household groups; solid lines denote interactions within households; dashed lines indicate relationships between household agents in two groups but in the same community; and the green circle indicates a community.

A feeling of risk among individuals is fueled by the type of information, the amount of information communicated, and the attention to specific information that may trigger fear and stimulate a learning process regarding a new response strategy [41]. Gained information helps individuals (i) to estimate the severity of the emerging event, (ii) to assess the probability of being exposed to infection, and (iii) to evaluate the efficiency of their coping responses. We used a complex network approach to illustrate the gradual processes from individual to collective learning in CABM (Fig 3). Each stage is presented as a single network over which a given learning process spreads. Each network in Fig 3 had the same set of nodes and connections to show how different processes can lead to different outcomes in the same network structure when different information is used to make decisions.

In individual learning (Fig 3, Process 1a and Process 1b), agents depend on their prior knowledge (memory, experience, and/or the perceived risk of the environment, such as visual pollution). Such learning is the process of gaining skills or knowledge, which an agent pursues individually to support a task [42]. **Group learning** is the process of acquiring new skills or knowledge that is undertaken collectively in a group of several individual agents and driven by a common goal [32]. Group learning can be realized by making all group members use their own ML algorithms to gather information to perform a specific sub-task (**decentralized**), and then pool their opinions collectively by making one decision for the entire group (Fig 3, Process 2a and Process 2b). Here, we adopt a “majority vote” as the resolution mechanism in the decentralized group decision-making. However, group learning can also be realized by introducing a single agent (**leader**) who uses ML to learn for the whole group to help it accomplish its group task (**centralized**). In the centralized group learning, agents in the group copy the decisions of their leader. In both cases, all agents that belong to a group share the same decision, but the information on which this decision is based on varies considerably (Fig 3, Process 3a and Process 3b).

Both individuals and groups may learn by either by taking information from their social networks (i.e., have it as an additional source of information in their ML algorithms) or not. When individual agents are **isolated** learners (Fig 3, Process 1a), they do not have a social network but use only their own information to make a decision in an isolated environment using the information they possess. When individuals learn in an **interactively** (Fig 3, Process 1b), they gain new skills or knowledge by perceiving information, experience, and the performance of other agents through their social network. Like individual agents, groups can also learn in isolation or interactively. In **isolated learning**, agents learn independently within their groups, without exchanging any information with each other or with their neighbors (Fig 3, Process 2a and Process 3a). In **interactive learning**, agents communicate with their neighbors to learn effectively within their groups (Fig 3 Process 2b and Process 3b). Neighbors could be members of the same group or belong to other income/education groups but live in the same community and share the water collection points. Therefore, there might be communication across the groups (Fig 3, Process 2b and Process 3b).

Groups can be defined in different ways and at different hierarchical levels. This model uses three levels of an organization, the **individual agent**, **groups of agents** and **communities** that comprise several groups. In CABM, household agents living in the same community are grouped based on their income and education level since their coping behavior depends on these factors. Agents’ behavior in the disease ABM also is contingent on their geographic location. Hence, all neighbors that share the same water fetching point may contact and exchange information between their groups in CABM.

The size and compilation of the groups impact the results of the different learning strategies. When applying interactive learning, a group’s decision can be influenced by information retrieved from neighbors inside the group and neighbors outside the group but inside the community. For interactive groups, Process 2b (Fig 3) shows a situation in which individual household agents make decisions that account for interactions in their social networks (as in Process 1b). Then each household conducts a majority vote, allowing it to proceed with the option chosen by the majority of its members. Process 3b (Fig 3) shows a situation in which the leaders of each group make decisions based on their interactions with others (nodes A, B, and C are leaders of groups G1, G2, and G3 respectively in Fig 3). The decisions of group leaders are adopted by the household agent of the group.

### 2.3 Simulation scenarios: Individual vs group learning

**Fig 4:**
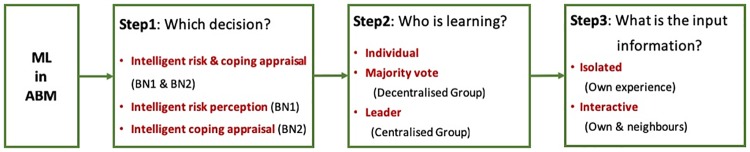
From individual to collective intelligence in ML-based ABMs.

We designed eight simulation scenarios to answer the research questions about the influence of isolated vs interactive individual learning (RQ1); centralized vs decentralized learning in processes–during both the risk perception (RP, BN1) and coping appraisal (CA, BN2) processes (RQ2); and collective learning about risk perception combined with individual coping appraisal (RQ3) on the dynamics of the epidemic and the performance of the model (Table 1). We systematically vary CABM settings following the steps in Fig 4 to change the gradient of intelligent learning (Steps 2 and 3) in different cognitive stages corresponding to our decisions of interest: risk and coping appraisal (Step 1).

**Table 1:** Simulation scenarios.

Model scenario	Decision that relying on ML	Agent that employs ML	Isolated vs interactive	Commentary
M1: **RP&CA (In-I)**	RP and CA (BN1 & BN2)	Individual (In)	Isolated (I)	An individual uses ML to update her risk perception and to take protective actions only based on her individual experience, neglecting any communication with others (Fig 3 Process 1.a)
M2: **RP&CA (In-N)**	RP and CA (BN1 & BN2)	Individual (In)	Interactive with neighbors (N)	An individual uses ML to update her risk perception and to take protective actions based on her individual experience as well as based on past disease experiences of peers (Fig 3 Process 1.b).
M3: **RP&CA (D-I)**	RP and CA (BN1 & BN2)	Majority vote (D) (decentralized group)	Isolated (I)	All agents in a group use ML to make decisions without taking the experience of others into account. The final decision on RP and CA is defined through the majority vote (Fig 3 Process 2.a).
M4: **RP&CA (D-N)**	RP and CA (BN1 & BN2)	Majority vote (decentralized) (D)	Interactive with neighbors (N)	All agents in a group use ML to make decisions taking the experience of others into account. The final decision on RP and CA is defined through the majority vote (Fig 3 Process 2.b)
M5: **RP&CA (L-I)**	RP and CA (BN1 & BN2)	Leader (L) (centralized group)	Isolated (I)	Each agent group randomly chooses a leader who uses ML to make a decision. The leaders decide in isolation without communicating with others; all group members mimic their decisions (Fig 3 Process 3.a)
M6: **RP&CA (L-N)**	RP and CA (BN1 & BN2)	Leader (L) (centralized group)	Interactive with neighbors (N)	Each agent group randomly chooses a leader who uses ML to make a decision. The leader considers the disease experience of others in the group and outside; all group members mimic leader’s decisions (Fig 3 Process 3.b).
M7: **RP(D-N), CA (In-N)**	RP (BN1) as in M6 CA (BN2) as in M2	RP: Majority vote (D) (decentralized group) CA: Individual (In)	For both RP and CA: Interactive with neighbors (N)	Taking the experience of others into account, all agents in a group use BN1 to decide on disease risks. The group members vote to evaluate the final risk perception for all group members (RP as in Fig 3 Process 2.b). Everyone individually assesses their own self-efficacy regarding disease prevention actions (CA). They run BN2 while considering past experience of others (CA as in Fig 3 Process 1.b).
M8: **RP(L-N), CA (In-N)**	RP (BN1) as in M4 CA (BN2) as in M2	RP: Leader (L) (centralized group) CA: Individual (In)	For both RP and CA: Interactive with neighbors (N)	Each agent group randomly chooses a leader who uses ML to decide whether the disease risk is real (RP). The leader considers disease experience of others in and outside the group; all group members mimic the leader’s RP decision (RP as in Fig 3 Process 3.b). Everyone individually assesses their own self-efficacy regarding disease prevention actions (CA). They run BN2 individually while considering the experience of others (CA as in Fig 3 Process 1.b).

Table 1 shows the setup of the eight scenarios that reflects the three stages shown in Fig 4.

### 2.4 Models setup and output measures of the cholera ABM

The area of the case study captured in CABM is 19.2 km^2^ and comprises of 21 communities. We assumed that high-income households bought water, so they were excluded from intelligent learning. Communities can have up to four groups based on their income and education levels. Ten to fifteen percent of the household agents in the case study area usually fetch water from the river. Two communities in our dataset (#11 and #20) hosted only high-income households, so they were excluded from the intelligent learning. Hence, we simulated 76 groups spread over 19 communities. Each simulation was run for 90 days with a time step equal to one hour. Given the inherent randomness of ABMs, we ran each model for 100 times, generating a new synthetic population every 10 runs.

Besides the extensive GIS data and aggregated data on disease dynamics, we ran a survey via a Massive Open Online Course (MOOC) Geohealth in two rounds (2016 and 2017) to gain data on individual behavior. The participants–primarily students from developing countries–were introduced to the problem of cholera disease, saw pictures of water, and were asked if they would use the water as it is (D1 in Fig 1), walk to a cleaner water point (D2), or use the water after boiling it (D3). The survey data were used to construct and train our BNs [36]. We also used these data to evaluate the results of expert-driven BNs in CABM [43]. Table 2 shows that trust in boiled water was much higher than trust in un-boiled water. Agents also changed their behavior and began boiling water in the model.

**Table 2:** Percentage of individuals decision type in both survey and CABM.

Decision type	MOOC (all participants)	MOOC (Participants from Africa)	CABM (average percentage of 100 runs)
No Risk—Use this water (D1, Fig 1)	42%	56%	42%
RP—Walk to another water point (source) (D2)	84%	77%	30%
RP—Boil water (D3)	72%	75%	57%

To evaluate the impact of individual and social intelligence on agents’ learning processes regarding risk perception and coping appraisal and the resulting patterns of disease spread, we used four output measures: **disease diffusion**, **risk perception**, **spatial patterns**, and **model performance**. These aspects are described in more detail in the ODD protocol (S1 Appendix).

We also measured the performance of models M1 –M8 in terms of run time and the number of intelligent decision steps, i.e., when agents called their BN1 and/or BN2.

## 3 Results and discussion

Given the stochastic nature of ABMs, we ran each of the eight models 100 times. The average and standard deviations of the results of these runs for each output measure were listed in Table 3.

**Table 3:** Output measures of the eight scenarios.

Model Scenarios		Output Measures						
		Duration (days)	Total of infected cases	Peak day -Epidemic	Peak value—Epidemic	Peak day—Risk perception	Peak value—Risk perception	SpI***
**Real data (2005)**		75	1621	42	181	N/A	N/A	1
**M1: RP&CA (In-I)**	Mean**	55	2,457*	35	232	88	501	0.65
	*SD*	2	195	1.3	30.12	1.9	103	
**M2: RP&CA (In-N)**	Mean**	68	2,279*	35	209	38	481	0.66
	*SD*	0.6	113	0.96	18.4	2.3	98	
**M3: RP&CA (D-I)**	Mean**	58	3,355*	37	345	90	501	0.62
	*SD*	3	402	2.5	83.2	0.4	233	
**M4: RP&CA (D-N)**	Mean**	55	3,046*	36	320	85	708	0.61
	*SD*	1.8	268	0.97	60.4	1.7	265	
**M5: RP&CA (C-I)**	Mean**	79	2,851*	37	215	44	676	0.7
	*SD*	2.13	243	1.5	26.8	1.2	114	
**M6: RP&CA (C-N)**	Mean**	79	3,071*	38	210	44	456	0.64
	*SD*	3.8	105	2.4	41.7	0.96	198	
**M7: RP(D-N), CA (In-N)**	Mean**	77	2,911*	37	307	89	610	0.61
	*SD*	1.65	78	1.62	14.5	0.92	122	
**M8: RP(C-N), CA (In-N)**	Mean**	75	2,107*	37	136	44	462	0.75
	*SD*	0.64	129	1.6	22	1.2	221	

(*) representing 57% of total infected cases

(**) the mean value is estimated across 100 simulations under different random seed for each scenario M1 –M8.

(***) Spl is spatial distribution of infected cases in both real dataset and the outcomes of the simulations (S1 Appendix).

Behavioral changes can lead to different duration times of the epidemic and reduce the number of infected cases [44]. This was shown by running CABM with the eight models. Models M5, M6, M7, and M8 recorded a longer duration of active infection during the epidemic (75–79 days, Table 3). These results are closer to the real duration of the epidemic in 2005 (75 days, Table 3). M5, M6, and M8 applied centralized learning, while M7 applied decentralized learning, but only for the risk perception stage. M2, which is individual learning with social interactions, also recorded a shorter duration when compared to the real data of 2005 (68 days in M2). However, isolated learning and decentralized learning for both risk perception and coping appraisals recorded shorter epidemic duration, with an average difference of -25% compared to the empirical data (Table 3).

All eight scenarios generated more infected cases than the empirical data. This was because infection with cholera bacteria leads to a clinical spectrum that ranges from asymptomatic cases to symptomatic cholera cases. Asymptomatic cases are not reported, although they represent roughly half of all cases [45]. In our simulations, we did not differentiate between symptomatic and asymptomatic cases; we considered all infected cases are considered to be symptomatic cases. Therefore, following [45], in Table 3, we reported that 57% of the total infected cases occurred when running the eight models.

M8, which uses centralized learning for risk perception and individual interactive learning for coping appraisal, reported the fewest infected cases (2,107 against 1,621 in reality). This was followed by M2 (individual social learning) with 2,279 cases and M1 (individual isolated learning) with 2,457 occurrences. These three values reflect the fact that when household agents learned to cope and make decisions individually, they were more efficient than when they were in groups. When these decisions were combined with social interactions, they lead to better protection (M2 and M8). In general, group behavior had a negative effect, although centralized groups had a less negative impact compared to decentralized ones. Finally, in M7, where household agents learned risk perception in decentralized groups and learned to cope individually, 2,911 infected cases were recorded (Table 3). Hence, CABM household agents’ engagement in decentralized groups for appraising disease risk hindered the perception of risk, lowering agents’ motivation to change their behavior to more protective alternatives.

The spatial distribution of infected cases (SpI) of M8 reported the closest SpI over the communities (0.75) compared to 1 in the empirical data. This was followed by M5, with 0.7 (Table 3). The spatial patterns of the two collective learning models (M8 and M5) reflected their similarity to the spatial patterns in the empirical data.

The correlation between the peak of the epidemic and the peak of risk perception reflects the responsiveness of the household agents’ risk perception of the epidemic. Scenarios M2, M5, M6, and M8 were more responsive. That is, the peak of risk perception in M2 came three days after its epidemic peak, and the peaks in M5, M6, and M8 came seven days after their epidemic peaks (Table 3). M1, M3, M4 and M7 showed peaks for risk perception near the end of the simulation time. Individuals in M1 were isolated, along with individuals in M3; therefore, they kept following their usual behavior of fetching water and using it as it is. In M4 and M7, household agents depended on majority votes in their groups to make their decisions on risk and to change behavior. More explanations are represented visually in the next sections.

Table 4 shows the number of steps and the time required to run one simulation of each model. The number of agents that were supposed to go for risk perception daily was 15% of the total number of household agents (which totaled 8,500). This percentage was derived from national statistical data from Ghana Statistical Services [35]. Over the 90 days of the epidemic, 114,750 agents appraised their risk perception (use their BN1). Table 4 also shows the number of steps, during which agents perceived the risk of disease (i.e., risk perception equals 1). Notably, in M3 –M8, if a group at large assessed the risk perception as zero, then none of its members did the coping appraisal, i.e., the number of steps when BN2 was activated is zero. In such cases, only the total number of steps with activated BN1 assessing risk perceptions was included in Table 4.

**Table 4:** Calculation of time and number of steps each model requires to run one simulation; agents in M3, M4 and M7 make decision twice (individually then within their group) which costs extra steps (two steps per day for M3 and M4 and one’s step for M7).

Model	Votes /day	Vote per simulation (steps)	Risk Perception (steps)	Average of Agents with RP = 1 daily	Coping Appraisal (steps)	Total (steps)	Run Time (minutes)
**M1: RP&CA (In-I)**	0	0	114,750	239	21,510	136,260	85
**M2: RP&CA (In-N)**	0	0	114,750	299	26,910	141,660	95
**M3: RP&CA (D-I)**	2	180	114,750	206	18,540	133,470	90
**M4: RP&CA (D-N)**	2	180	114,750	352	31,680	146,610	125
**M5: RP&CA (C-I)**	0	0	6,840	410	6,840	13,680	26
**M6: RP&CA (C-N)**	0	0	6,840	260	6,840	13,680	35
**M7: RP(D-N), CA (In-N)**	1	90	114,750	318	28,620	143,460	75
**M8: RP(C-N), CA (In-N)**	0	0	6,840	293	26,370	33,210	45

Models with centralized learning required the shortest computation times (Table 4). For example, M5, where only the isolated leaders with the centralized learning consult their BNs, had the best performance with the shortest runtime. Moreover, M5 and M6 recorded the fewest steps across all models. Although the average number of agents with risk perception per simulated day was high (410 agents), there were only 6,840 steps in risk perception and the same number of steps when coping appraisal MLs were activated. That is, only leaders activated their BN1 and BN2. This is only 22% compared to what it would be if agents decided individually. Without voting, only one agent per group assessed the situation and made decisions. This made M5 and M6 time-efficient. On the opposite end, M4 recorded the highest computational time because of the intensive calculations required in the individual agents’ network and the decentralized group network.

Among all models, M4 recorded the longest process time. Agents individually perceived risk (BN1) before going back to their groups to negotiate a final decision on risk perception and then repeating the same individual-group sequence for the coping appraisal.

In models M5, M6, and M8 only one agent per group–a total of 76 leaders–assessed risk perception daily, leading to 6,840 steps over the 90-day epidemic. In M5 and M6 only the 76 leaders also went for coping appraisal, while in M8, the group members individually assessed the coping appraisal (26,370 steps in M8 vs 6,840 steps in M5 and M6).

Calibration of the original model was conducted in two steps: first the hydrological sub-model was calibrated, followed by a calibration of the complete model [35]. After the calibration, a stability check was performed [35]. For the current work, the objective of this epidemic model is not to reproduce the real data. It is focusing on the impact of social interactions (present or not, on the level of individual or groups) on both risk perception and coping appraisal of the individual agent. To calibrate a scenario further, one would need risk perception data for that area for the duration of the epidemic. However, such data are very scarce, not only for Kumasi but worldwide. Hence, risk perception was randomized at initialization. Therefore, the eight models cannot be calibrated individually because they need to be comparable at initialization.

S2 Appendix shows the statistical analysis that was performed on the output data of the eight models to show and analyze the distribution of the obtained results.

### 3.1 Making decisions individually does not pay off (M1 vs M2)

**Fig 5:**
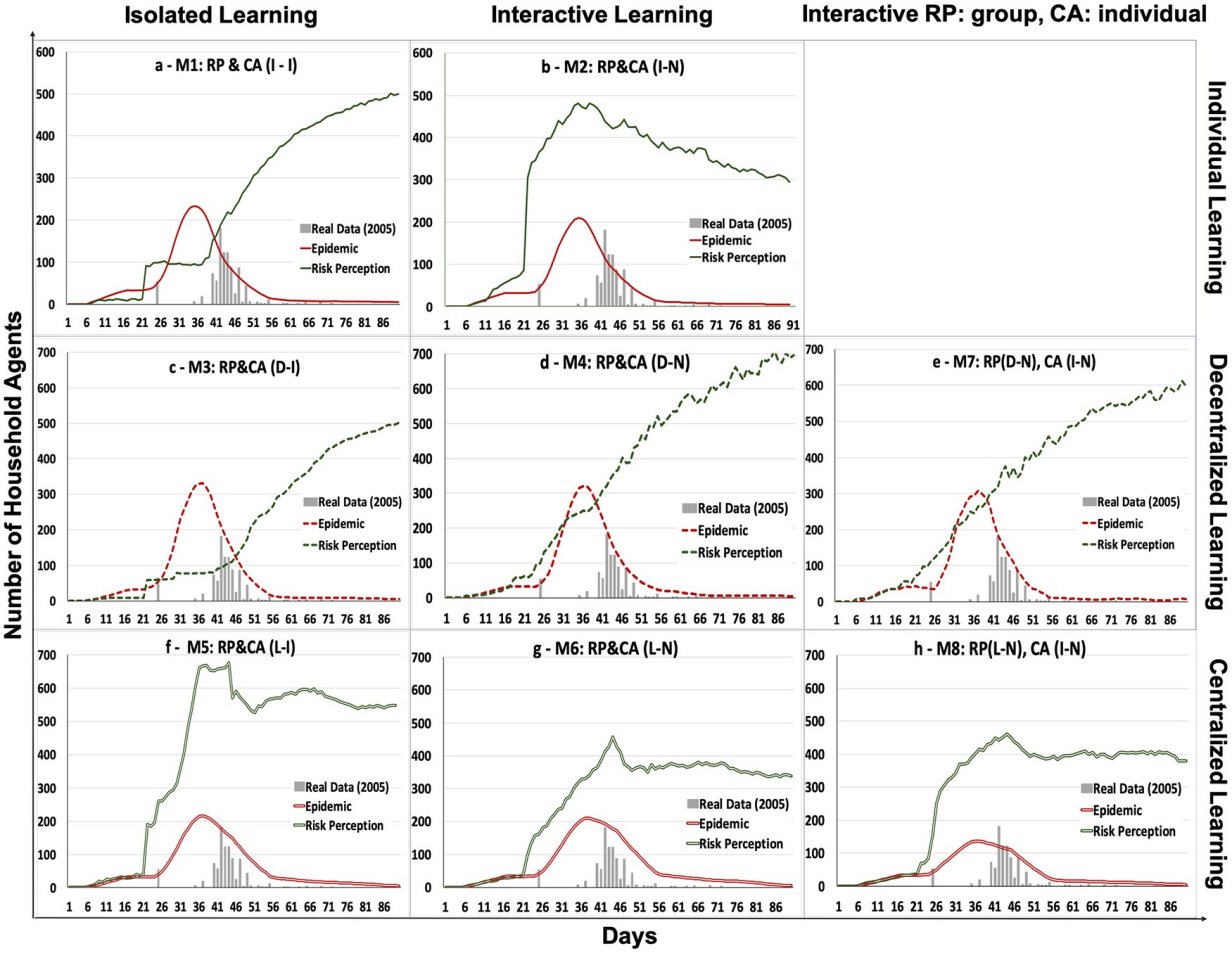
Epidemic curves (in red) and risk perception curves (in green) for scenarios M1–M8.

When household agents evaluated the risks of getting cholera and made coping decisions individually (M1), they relied only on their own experience. That is, each had individual BN1 and BN2 and did not communicate with neighbors. Scenario M2 extends this stylized isolated benchmark case by assuming that while agents continued to make decisions individually, they did share information with neighbors about the perception of risk and protective behavior. That is, both BN1 and BN2 included neighbors’ experiences among the information input nodes. Fig 5 shows the epidemic curves and the dynamics of risk perception for all scenarios.

In the absence of social interactions, more agents became infected with cholera. The peak of the epidemic curve in M1 (In-I) is higher than M2 (In-N), leading to 11% more cases of disease (Fig 5 and Table 3). Overlaying risk perception and epidemic curves suggests that when agents made decisions in isolation (M1: In-I), the dynamics of risk perception were hardly realistic (Fig 5a). Namely, when the epidemic was at its peak, household agents in M1 responded very slowly, with BN1 delivering a wrong evaluation of risk perception (Fig 5a). They became aware of the risks very late, so when the epidemic vanished, the number of agents with risk perception = 1 kept increasing. In the absence of communication and experience sharing among peers (In-I), the information about disease spread slowly and there was a significant time-lag between the occurrence of the disease and people’s awareness. The small stepwise increase, around day 21, was because the media started to broadcast information about the epidemic on that day.

In M2, household agents behaved according to the expected pattern: risk perception became amplified by media coverage and social interactions and then vanished as disease cases became rare (Fig 5b). Only those who experienced cholera infection in their households remained alert. Household agents in M2 after day 21 had more responses to the media’s news compared to isolated agents. Media supported the agents’ social interactions with their neighbors, which led to more agents perceiving risk, especially when the number of infected cases reached their peak (Fig 5b). Even in M2, there were limitations of making decisions about risk perceptions individually: risk perception fell too quickly, implying that people stopped worrying about the epidemics although they continued.

Since household agents in M1 did not have interactions with other agents, running this model required less time than M2 (creating a 10% increase in performance, Table 3). The interaction between household agents required time to process the information exchanged between agents.

**Fig 6:**
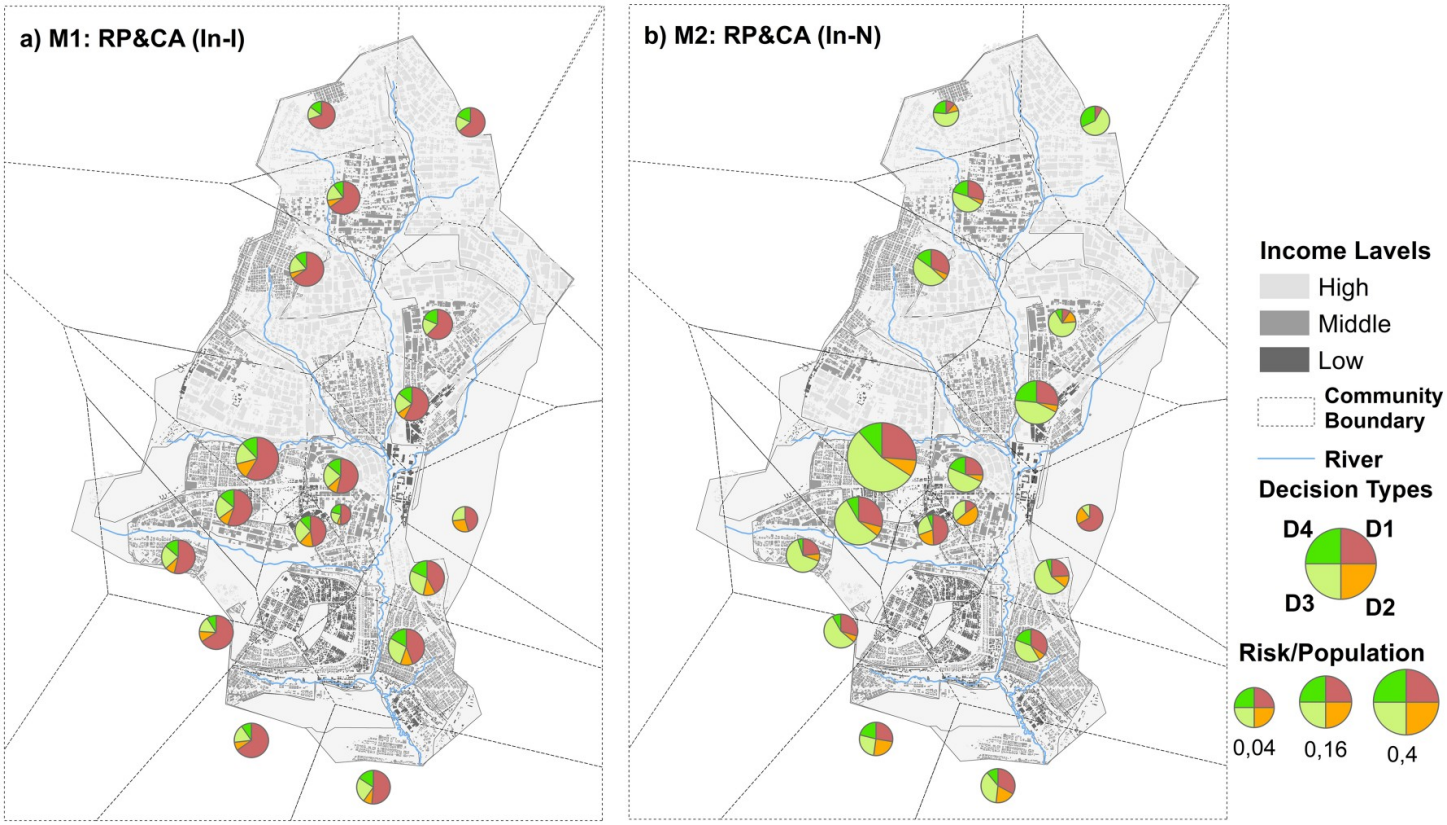
Spatial distribution of different coping appraisal decisions of scenarios M1 and M2; the size of the pie represents the size of household agents with risk perception = 1 over the community population.

In addition, (M1: In-I) and (M2: In-N) were approximately the same in terms of the realistic spatial distribution of infected cases over the communities, with values of 0.65 and 0.66, respectively (Table 3). Fig 6 presents the spatial distribution of decision types over the study area in both M1 (In-I) and M2 (In-N). The household agents in isolated learning were not aware of the cholera-infected cases in their neighbors’ household. Household agents in M1 took an unsecured decision and trusted more in using the water fetched from the river as it is (D1 in Fig 6a). Household agents in M2 were more rational and mostly boiled the water that they fetched from the river (D3 in Fig 6b).

### 3.2 Majority vote is imperfect (M3, M4, and M7)

In decentralized learning, groups of household agents vote for risk perception and coping appraisal. The final decision of the group is the output of the majority votes. Thus, all group members follow the final decision of the group. These groups represent the democratic system, which depends very much on the composition of the group. The decentralized groups with a majority vote can lead to a negative perception of risk. Besides, a coping appraisal that depends on a majority vote can lead to inappropriate decisions regarding protection from cholera. When individuals are engaged in social groups, their behaviors are not independent anymore [46]. This leads to an increase in the randomization of decentralized learning models (M3 and M4). These two models had higher standard deviations in all measures (Table 3).

The qualitative patterns of the three scenarios (M3, M4, and M7) were the same regardless of the social interactions that added new information to ML (Fig 5). For the development of the disease, the voting mechanisms seemed to overwrite individual judgments. The M3 scenario assumes that household agents were isolated when performing risk perception and coping appraisals. In contrast, M4 and M7 allowed household agents to communicate with neighbors during the process of risk perception and before making a coping decision. As a result, M4 and M7 generated greater risk perception than M3 (Fig 5c, 5d and 5e). This suggests that the social interactions still amplify both the awareness of risks and the diffusion of preventive actions.

Given approximately the same peak heights, the epidemic curves in the three majority voting scenarios reported more infected cases than the other models. Among the majority votes, M7 reported the fewest infected cases, since household agents in their coping appraisal relied on themselves rather than their decentralized groups. Overall, it seems that all three models–M3, M4, and M7 –got the process of disease risk evaluation wrong. In those cases, risk perception slowly grew in the days when the epidemic was peaking (Fig 5c, 5d and 5e) and did not react to the peak in any way, which is unrealistic. Moreover, risk perception in the three models continued to grow when the epidemics were almost over. Risk perception peaked when there was no longer a risk, i.e., in the last days of the simulation, as shown in Table 3. Hence, group voting on risk perception operated with a major time lag: household agents ignored early signals of disease that occurred in just a few households. Then they increased their awareness about risk only when most of them were already infected, and they continue to be falsely alerted when the epidemic was over.

In M3, the small stepwise increase in risk perception represents the response to media, and it is similar to M1 (In-I) in its development (Fig 5c). The household agents in their decentralized groups did not have contact with neighbors, therefore, no cases were reported to them from their neighborhoods. As such, they were disconnected from what is happening around them.

In M4 and M7, which included social interactions, the development of risk perception seems more responsive, especially after the activation of media on day 21. Nevertheless, their response time was still slow (Fig 5d and 5e). In these models, the group decisions were very much dependent on the composition of the group members’ opinions. These varied from one another and had different information sources for the final decisions about risk perception (in both M4 and M7) and coping appraisal (in M4).

**Fig 7:**
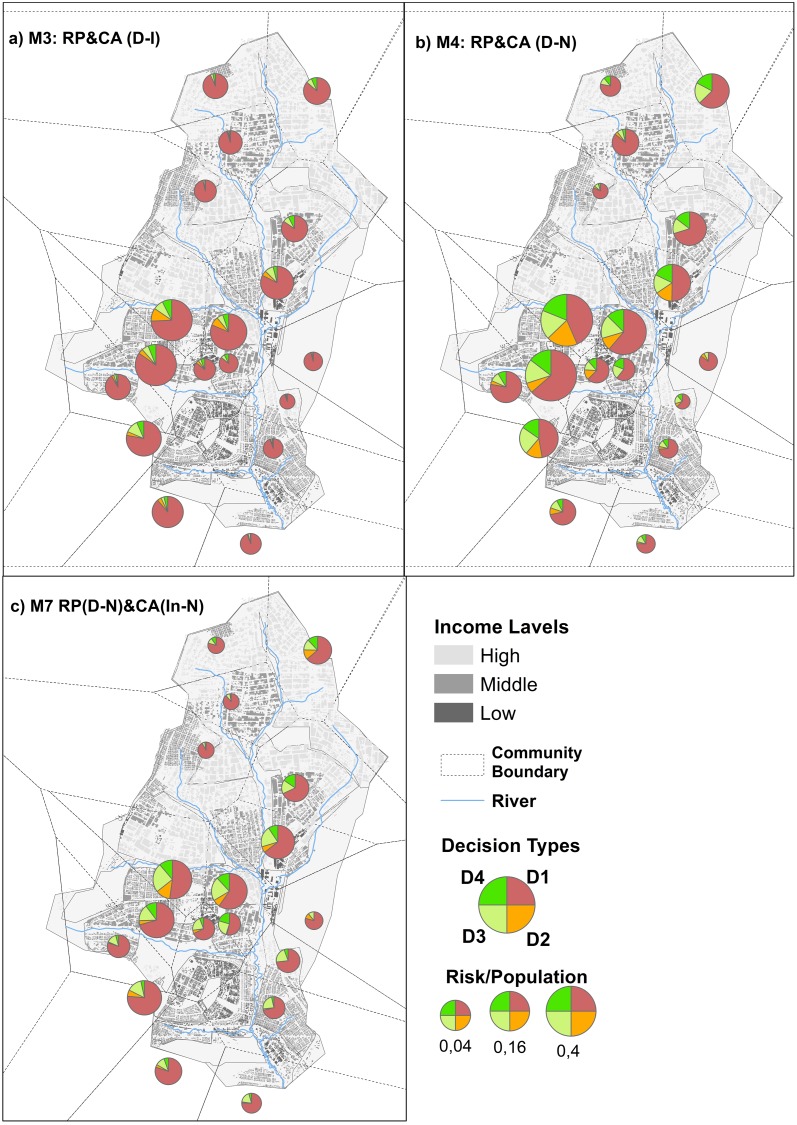
Spatial distribution of different coping appraisal decisions of scenarios M3, M4 and M7; the size of the pie represents the size of household agents with risk perception = 1.

Thus, majority voting led to unsecured decisions. Groups in these models were heterogeneous in that household agents had different levels of exposure to the group members with which they voted. Decentralized groups with isolated input information (M3) led household agents to vote to use the water fetched from the river (D1) most of the time (Fig 7, map a). Because of their lack of communication with neighbors, household agents missed the opportunity to get information about the infection in their neighborhoods. This explains the higher numbers of infected cases in the majority vote models.

Social interactions in both M4 and M7 helped agents make better decisions, although following the majority still biased their choices. For instance, in M4 high-income communities (upper communities in Maps b and c, Fig 7), household agents mostly used the river water as it was even though they were rich enough to boil it before using it (D3) or to buy bottled water (D4). The opposite also occurred when a majority vote forced low-income households to buy bottled water, which is an expensive decision for them. The group voting on the coping appraisal in M4 might have made individual members uncomfortable when they followed the decisions of their groups even though they might not protect. In reality, household agents sought a balance between preventive behavior and their capability to implement it. Moreover, there is always the possibility of routinely changing one’s mind based on daily updates of information regarding the epidemic and updates from neighbors.

As in M4, the household agents in M7 relied on their decentralized groups for risk perception. This, often led to risk ignorance (Fig 5e). However, since the agents in M7 decided on coping appraisals individually, more agents adopted D1 (Fig 7c). When they perceived risk during the last days of the epidemic, household agents in the middle-income level switched to boiling water or buying bottled water (D3, D4 in Fig 7c). Those in the low-income level walked to another water fetching point (D2).

### 3.3 Impact of leaders (M5, M6, and M8)

In centralized groups, one household agent is randomly selected to be the group leader. The leader is responsible for risk perception and the coping appraisal of the group. Group members copy the risk perception and disease preventive decisions of their leaders. It is argued that group leaders may improve their group’s performance if they model the responses to the situation the group faces [47]. In this article, we considered two types of leaders: a dictator making top-down decision about risk perception and coping strategy (M5 and M6), and an opinion leader evaluating risk perception top-down but giving group members the freedom to pursue their own disease coping behavior (M8). The qualitative trends of all three models coincided with what is expected: peaks caused by amplification of risk perception followed by a gradual decrease when epidemics plateau (Fig 5f, 5g and 5h). The centralized group learning on average represented the processes well, as the leader alerted the group members about the disease. However, since no real data are available on risk perception dynamics or the actual coping behaviors that people pursued during the epidemic, we cannot determine which of the models M5, M6, and M8 is the best. The following subsections compare models with a leader-dictator (M5, and M6) to one with an opinion leader (M8).

#### 3.3.1 As a dictator (M5 and M6)

A dictator-leader decides on behalf of his group regarding disease risk and coping strategies, and both decisions are adopted top-down. A dictator leader learns either in isolation (M5) or in interaction with her/his neighbors (M6). Isolated dictators in M5 are overestimated disease risks (Fig 5f). For example, if such a leader had his/her own bad experience with cholera, s/he would keep warning the group. With social interactions (M6), there is less uncertainty in the process of updating the risk perception than in M5. For example, compare risk perception assessments around the epidemic peak (Fig 5g).

**Fig 8:**
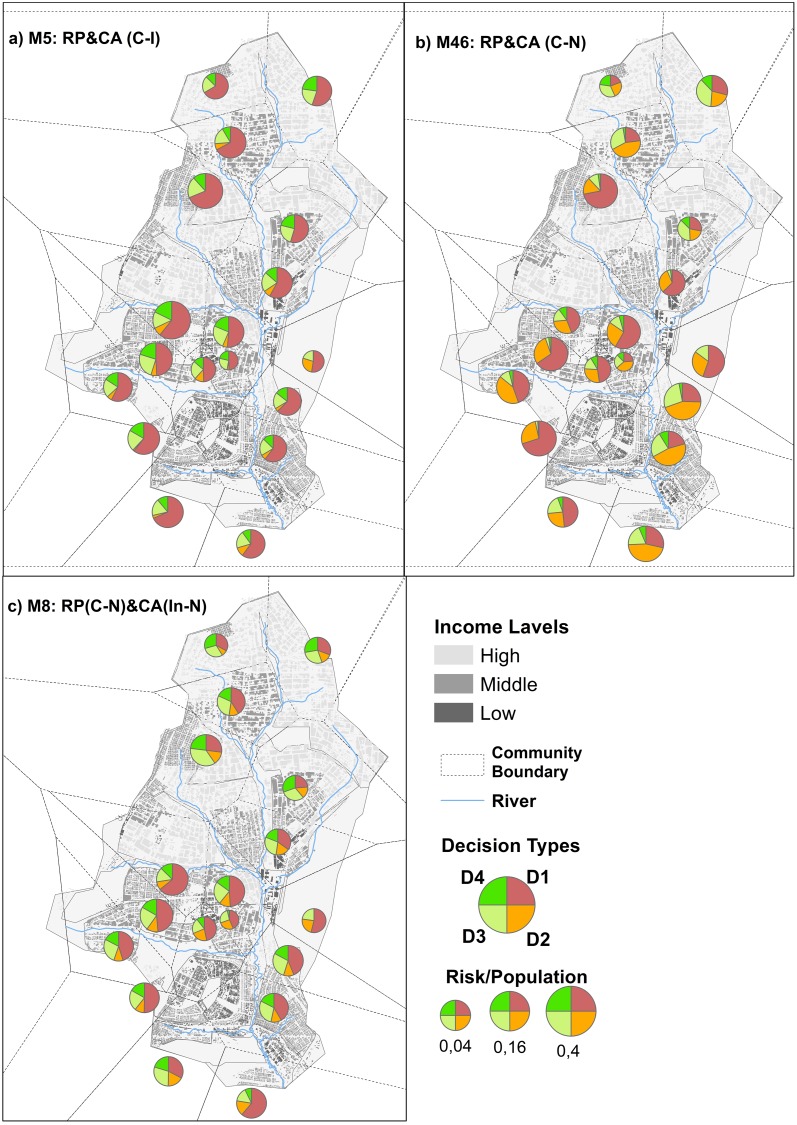
Spatial distribution of different coping appraisal decisions of scenarios M5, M6 and M8; the size of the pie represents the size of household agents with risk perception = 1.

Fig 8 illustrates the impact of social interactions on the dictator’s decisions regarding coping appraisal. Isolated leaders guided their groups to various types of decisions (Fig 8a), which were sometimes less secure decisions (e.g., D1). With social interactions, leaders relied on their neighbors and decided more often to walk to a point along the river where the water was cleaner (D2). Very few dictators directed their groups to boil the fetched water (D3) or buy bottled water (D4) (Fig 8b). This shows how centralized decisions making undermines heterogeneity in individual circumstances, such as disease exposure or coping capacity.

#### 3.3.2 As an opinion leader (M8)

In M8, the leaders in the centralized groups were responsible for evaluating disease risks for their groups, but they interacted with neighbors during the risk perception process. For the coping appraisal, the group members made their own decisions, using the information from their social networks. As a result of this combination of centralized speed alertness about risk perception and individual coping strategies, M8 generated the fewest infections. The shape of the epidemic curve (except for its height) is very close to the empirical data of 2005, (Fig 5h). As in M6, the uncertainty in the process of risk perception in M8, is lower than in M5 (Fig 5h). The risk perception curve developed around the epidemic peak followed the dynamics of the epidemic (Fig 5g).

When group members relied on social interaction to learn about the effectiveness of various coping strategies but eventually chose one themselves (M8), there was a diversity of coping strategies. Fig 8c shows the spatial distribution of different types of decisions during the simulation. More household agents went for D3 and D4, which were considered to be the most protective decisions. Consequently, communities pursued at least three types of decisions, reflecting the disease coping diversity so important for resilience.

## 4 Conclusions and future work

The goal of this paper is to perform a systematic comparison of individual vs group learning. The methodological advancements showed that different implementations of individual and collective decision-making in agents’ behavior led to different model outcomes. In particular, the stepwise approach of testing how learning (on a gradient from individual learning–without any interactions–to collective–with social networks) affects an ABM’s dynamics is generic and can be used for other models. To illustrate the subtle difference in implementing learning in ABMs, we used the example of the spatial empirical ABM of cholera diffusion with intelligent agents that employ ML to assess disease risk and decide on protective strategies, which define the dynamics of the epidemic. Interactive learning, which assumes that agents share information about risks and potential protective actions, outperformed isolated learning both for individuals and in groups. This underlines the fact that social learning in the decision-making process is very important in ABMs. While we used disease modeling as a case study, the results may be contingent on the endogenous dynamics of this particular cholera ABM. Notably, simulation results may differ for ABMs with other underlying dynamics. This calls for further scrutiny in testing and reporting cases of intelligent social and individual learning in other models.

The results indicate that decentralized groups with majority votes are less successful than groups with leaders, whether dictators or opinion leaders. When evaluating current disease risks, majority voting appears to be the worst mechanism for group decisions, often arriving at a wrong decision because of time lags compared to the dynamics of objective disease risks. Perceiving risk is a very personal decision-making process [48]. In contrast, when leaders develop risk perception and propose it to the group, such groups perform better in terms of risk appraisal. Moreover, opinion leaders are very effective in helping their group members be alert about disease while giving them the freedom to make coping decisions that accommodate heterogeneity in their socio-economic status and geographical locations. In contrast, dictator- leaders and majority votes that impose a decision that all group members must follow are less effective in reducing the incidence of disease.

In our simulation experiments, the structure of the groups is simple and is formed based on the spatial and socio-demographic characteristics of the agents. As grouping seems to have an impact on the spatio-temporal diffusion of the disease, the importance of disease modeling stresses the fact that for this type of model a careful evaluation of the social structures in the case study area should be conducted, to generate trustworthy results. Future research should focus on constructing groups based on different variables (family ties, religion, tribes). Also, in our ABM the leaders had no particular knowledge but were randomly selected and assigned to groups. In reality, this may not be the case. Leaders may have access to better information or have already earned the group’s trust and respect. In addition, decentralized groups can be improved by giving greater weights to more trusted partners to make wise decisions.

The model’s performance can be a strong argument when the number of agents is massive, e.g., when simulating a pandemic or epidemics within a very large population is needed to detect a worldwide diffusion mechanism. In that case, social group learning, as described in model M5, is a very good alternative to individual interactive behavior. Moreover, M5 shortens the computation time by 73% while maintaining a good quality model output.

The number of contacts each household agent has when they are in their collective learning may impact the diffusion of cholera. However, running a fat tail distribution of the number of contacts would be an interesting topic for future study.

Different considerations steer the ultimate decision on which type of social behavior to use. Besides the technical model performance metrics discussed here, the choice of a particular type of social behavior can also be based on the society that is being modeled. Different political systems, the presence of tribes, and different ethnic groups or religious leaders require careful considerations of the social interactions in a model. One should make sure that the actual situation regarding social learning represents the cultural and social norms of the society being modeled.

In this article, it was not possible to define, which implementation (M1 –M8) represented the situation in Kumasi most closely. To validate the risk perception-behavior, one would need risk perception data for that area for the duration of the epidemic. However, such data are very scarce, not only for Kumasi but worldwide. As we illustrated in this study, many different implementations of social behavior using ML are technically possible, but data are needed to validate alternative implementations. Yet, research on risk perception during epidemics is often conducted too late (when the peak is over) or at distance (not in the area where the disease spreads). Hence, researches provide little empirical proof of people’s behavior and risk perception. More research on risk perception during epidemics, including other variables such as cultural aspects and group behavior, can be very helpful in generating a model that represents a specific society realistically.

On a technical note, agent-based modeling software does not always include ML toolkits and libraries. This complicates the implementation of different types of social intelligence. Hence, better integration of ABM and ML in one software package or linkable libraries could eliminate this problem in the future.

Finally, an important direction of future research is to implement other ML techniques besides BNs, such as decision trees and genetic algorithms. In addition, modeling groups with different ML algorithms may lead to different results since groups will be heterogeneous in terms of members’ learning algorithms. Several developments in health research drew our attention to the implementation of learning in disease models. One is the impact of fake news on the behavior of people. The other is the fact that human behavior toward vaccination can change radically based on (fake) news it. Therefore, including these factors and testing their impact on the behavior of agents may lead to more conclusions for policymakers to consider in their efforts to control epidemics.

## Supporting information

S1 Appendix Cholera ABM (CABM) in ODD protocol. (DOCX)

S2 Appendix Normality test. (DOCX)
