# Supplementary material for: Risk perception and behavioral change during epidemics: Comparing models of individual and collective learning
Source: PLoS One. 2020 Jan 6;15(1):e0226483. doi: 10.1371/journal.pone.0226483 (PMC6944362; doi:10.1371/journal.pone.0226483)
Supplement: S1 Appendix — (DOCX) [file pone.0226483.s001.docx]

# S1 Appendix: Cholera ABM (CABM) in ODD Protocol

## Overview

### Purpose

This model was initially developed to check if runoff water from open dumpsites in Kumasi Ghana can explain the diffusion of cholera during an epidemic in 2005 [1]. The basic model has been adjusted to include risk perception using Bayesian networks by Abdulkareem et al. [2]. In both previous implementations, agents take decisions individually and no group processes are implemented. The purpose of this research is to investigate if different types of group behavior and variation in the level of interaction between agents influence the cholera diffusion patterns.

### Entities, State Variables, and Scales

The model consists of four sub-models: a disease model, a hydrological model, an activity model and a visual pollution sub-model. The hydrological model take care taking care of the flow of rainwater over the land surface and the collection of this water into a river. This model simulates the infection of river water with cholera. The activity model, which drives the collection of water by agents (leading to possible infection) and the dumping of faecal materials on open dumpsites (driven infection back to the environment). It also evaluates possible risk (risk assessment) and, in case risk, the choice of of using an alternative water source (coping). The Visual Pollution sub-model calculates the level of visual pollution at water collection points which is used as a proxy for the safety of the river water. A complete overview of all agents and their attributes is provided in S1 Table 1.

S1 Table **1**: Attributes and behavior of agents in CABM

| Agent name | Attributes | Behavior |
| --- | --- | --- |
| Household | - Income level - Hygiene level - Water collection point - Access to tap water - Education level - Memory | - Fetching water - Dump waste - Perceiving risk - Make coping decisions - Evaluating pervious decision - Fetch water (new timing) |
| Individuals | - Household ID - Gender - Blood type - Age - Health status | - Use the water (drink, cook) - Infected/recovered |
| Rain particles | - Infection level - Volume - Travel time |  |
| Media^*^ | - Cholera News | Broadcasting cholera news |

All environments are static except the water collection points, which vary in infection level during the simulation runs (S1 Table 2).

S1 Table **2**: Environment Elements in CABM

| **Submodel** | **Environment** | **Function** |
| --- | --- | --- |
| **Initialization** | - Boundaries of income levels | - Used during the creation of the synthetic population and distribution of agents over the study area. |
|  | - Community boundaries | - Thiessen polygons were drawn as community boundaries, in which household agents of the same community, income-level and education level formulate their groups |
| **Hydrology** | - Elevation layer (DEM) | - Used to calculate runoff |
|  | - River | - Used for the collection of water |
| **Activity model** | - Water collection points | - Have infection level. - Places where people collect river water |
|  | - Residential locations | - Represent housing block. |
|  | - Dumpsite locations | - Use by households to dump waste. They can become infected with cholera. - Influences visual pollution. |
| **Visual Pollution** | - Dumpsite locations | - Garbage flow from the open dumpsites to the river. |
|  | - Water – collection points | - Household agents evaluate the visual pollution of water collection points during the risk perception process |
|  | - Elevation layer (DEM) | - The flow of the garbage in the river water is calculated based on the DEM. |

The case study area captured in CABM covers 19.2 km^2^ and consists of 21 communities. The spatial resolution is 30 by 30 meter; and the simulation time step is one hour. We run the model for 90 days.

### Process Overview and Scheduling

The processes included in the CABM model are illustrated in S1 Fig 1 and described below.


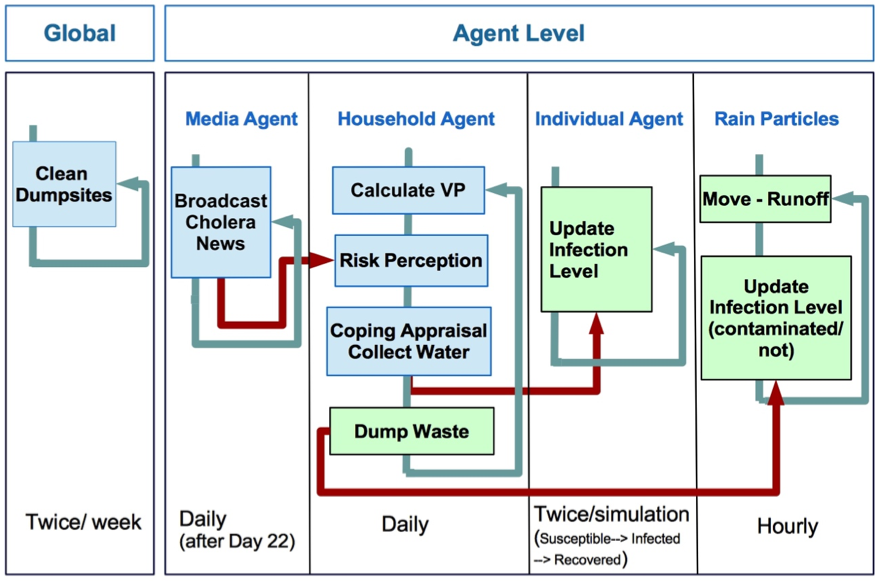


**S1 Fig 1: CABM Processes Overview; blue boxes refer to these activities that are related protection procedure and green boxes refers to these activities that are related to getting cholera infection. Red arrows refer to the impact of activities of a certain agent type on another agents; while blue arrows refer to repeating activities by a certain agent**

When **household agents** need to collect water from the river, they determine the pollution level around their water collection point and collect information from their neighbors, determine the media value and check their memory (Calculate VP – S1 Fig 1). When they determine risk, they proceed to the Coping Appraisal (Coping Appraisal Collect Water – S1 Fig 1). Household agents perceive risk daily – unless they have a household member infected with cholera.

The **individual agents** update their infection Level (Update Infection level – S1 Fig 1) daily, following the Coping appraisal and Water Collection phase.

The **media agent** can be scheduled to start broadcasting news about cholera from a particular day. After activation, the broadcasting will continue until the end of the simulation run.

The **rain particles** move over the terrain (DEM), updating their position every hour (Move – Runoff – S1 Fig 1). They can become infected (from infected dumpsites) and carry this infected to the nearest river or tributary while flowing over the terrain. This will lead to infected water collection points. The rain particle infection level is updated hourly.

The **dumpsites** are a source of cholera infection. They become infected by households that are dumping infected faecal materials (Dump waste – S1 Fig 1). Twice a week, dumpsites are emptied. Removing the garbage from dumpsites has an impact on the spread of cholera and influences the visual pollution level of the river water points when household agents evaluate the risk.

## Design Concepts

### Theoretical and Empirical Background

The behavior of people has an impact on the chance they have of getting ill. By perceiving the risk of infection, a person can decide to take precaution measures to lower this risk. In this model, we use the Protection Motivation Theory (PMT) as the theoretical framework for risk perception. PMT was initially proposed by Rogers in 1975 and was revised in 1983 to contribute conceptual visibility to the understanding of fear appeals [3]. PMT splits the reaction of a person to a dangerous situation in two steps: “threat appraisal” and “coping appraisal”. It takes information from various sources as inputs and delivers a decision on what action to take as on output (S1 Fig 2).


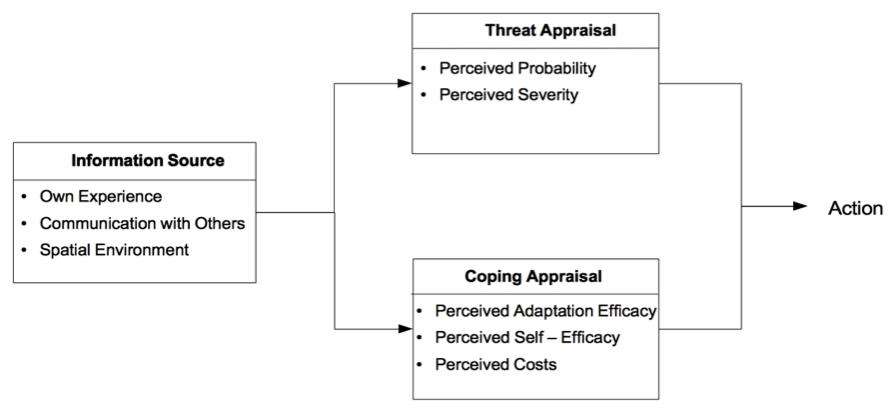


**S1 Fig 2: Cognitive Progress of Protection Motivation Theory (PMT)**

In the **threat appraisal** **stage** (risk perception), an individual assesses the probability and consequences of a risk event occurring, to judge how seriously s/he has to take the threat, while the perceived probability measures how susceptible the person is to the communicated threat. The purpose of this stage is to detect whether the risk is at an acceptable level or not.

In the case of perceived risk, the person is motivated to adopt the protective behavior by passing to the coping stage. The **coping stage** consists of two main parts adaptation (response) –efficacy and self-efficacy. Adaptation-efficacy measures the effectiveness of protective behavior against the harmful situation – i.e. the beliefs of a person that the recommended behavior will protect him/her. While the self-efficacy measures the ability of a person to perform the recommended behavior. Besides, the person needs to evaluate the cost required to cope with the threat. This involves the psychological, physical and social consequences for the person’s adaptation process.

### Decision Making

Household agents with low or middle income and no access to tap water have to look for another source of water. Usually, this source is the river. The perception of risk might trigger these agents to assess the state of the river water. The assessment is done through observing the visual pollution at water collection points and through contacts with neighbors sharing this water collection point. Also, the Media’s reports are taken into account. This assessment leads to the use of cleaner water to avoid cholera. The decisions can be any of the options: buying water, boiling water, using the water from the nearest water collection point, or walking to upstream water collection points that are closer to a spring.

### Learning

We implemented learning according to the implementation strategy described in [4] as shown in S1 Fig 3:


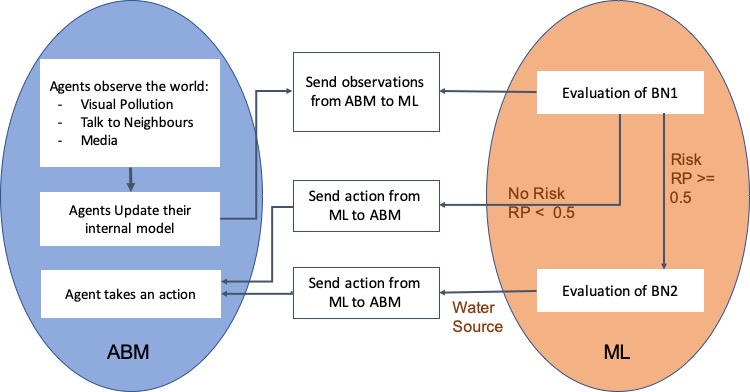


**S1 Fig 3: the implementation of the ML algorithm and the ABM**

The AI technique, Bayesian Networks (BNs), was implemented to operationalize the threat appraisal and coping appraisal of the PMT framework. In our model, variables have a finite set of discrete states. In this model we used two BNs – BN1 to model the threat appraisal and BN2 to model the coping appraisal.

In this section, we first discuss the implementation of the threat appraisal and coping appraisal via Bayesian Networks (S1 Fig 4).

Both BN1 and BN2 consist of several nodes connected to evaluate both risk perception and coping decision respectively. Each node represents a variable that can impact the agents’ decision process. The design of the BNs is based on expert information and the BNs were initialized based on expert knowledge and data gathered from two online surveys [5]. The expert knowledge drove the structure of the BNs while the values of nodes and the conditional probability table (CPT) were driven from the literature, census data of Kumasi, Ghana and the survey data. The BNs are trained during the simulation of the CABM.


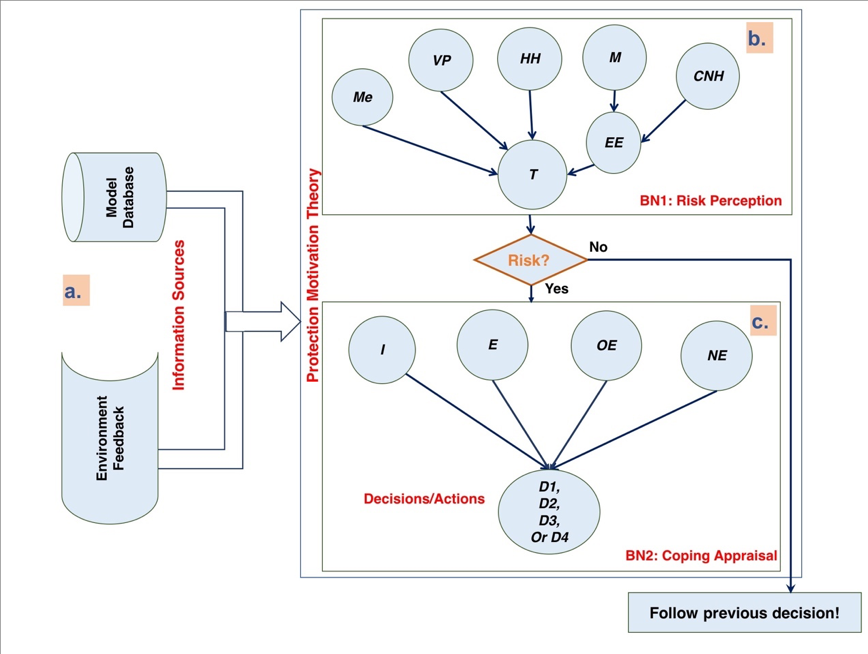


**S1 Fig 4: Implementation of Agents' cognitive model (PMT) using two Bayesian Networks: BN1 and BN2 [2]**

### BN1: Threat Appraisal

Household agents with a low- or medium-income level without access to tap water need to fetch water from the river. Before an agent in the ABM decides to use the river water, the agent consults BN1 to determine if there is a risk. The following values are communicated to the learning algorithm (S1 Fig 4): memory value (Me), visual pollution of the water collection point (VP), household (HH) health status, Media (M) and communication with neighbor households (CNH). Media and communication with other households are combined into “epidemic evidence” (EE) which indicates the total number of disease cases the agent is aware of outside their household. The epidemic evidence (EE) differs per agent as the household situation of the agents varies.

The uncertainty and reasoning of the risk perception are governed by rules that can easily be formalized using formula (1). For example, we have the states {yes, no} for household health status (HH), {yes, no} for Threat (risk perception), then the formula of connecting these two variables according was designed as:

|  | $\mathbf{P} \left( \boldsymbol{T}_{\boldsymbol{\{yes, no\}}} \vert\boldsymbol{HH}_{\boldsymbol{\{yes,no\}}} \right)\boldsymbol{=}\frac{\mathbf{P} \left( \boldsymbol{HH}_{\boldsymbol{\{yes, no\}}} \vert\boldsymbol{T}_{\boldsymbol{\{yes, no\}}} \right)\mathbf{P} \left( \boldsymbol{T}_{\boldsymbol{\{yes, no\}}} \right)}{\mathbf{P} \boldsymbol{(}\boldsymbol{HH}_{\left\{ \boldsymbol{yes, no} \right\}}\boldsymbol{)}}$ | (1) |
| --- | --- | --- |

This was also applicable for computing the probability of Threat based on Visual Pollution, and Memory as both variables with states {no, low, high} and {yes, no} respectively. We evaluated the epidemic evidence (EE) from what household agents hear via their communication with other agents and the media agent. As claimed by Bayesian rules, the prior probabilities of the nodes should be specified in order to obtain the posterior probabilities. These prior probabilities represent an integral part of humans explaining the certainty. The final formula for the Threat (risk perception) node will be calculated as:

|  | $\boldsymbol{P} \left( \boldsymbol{T} \vert\boldsymbol{Me, VP, HH,EE} \right)\boldsymbol{=}\frac{\boldsymbol{P} \left( \boldsymbol{Me, VP, HH, EE} \vert\boldsymbol{T} \right)\boldsymbol{P} \boldsymbol{(T)}}{\boldsymbol{P(Me, VP, HH,EE)}}$ | (2) |
| --- | --- | --- |

The output of BN1 is a value between zero and one that represents the probability of low or high-risk perception. We assume the agent is at risk if the probability of risk perception is greater than 0.5 (high or “1”). When at risk the agent will proceed to coping appraisal. If not at risk the agent will keep on using the same water as in the previous time step.

### BN2: Coping Appraisal

In case of perceived risk an agent may: still use the polluted water (D1), walk (find another location to fetch water) (D2), boil the fetched water (to increase the safety) (D3), or buy bottled water (D4). In order to make one of these four decisions, a number of values for the nodes (variables) are required: the income level of the agents (I) (either medium or low); their education level (E) (either educated or uneducated); and the feedback on theirs (OE) and their neighbors’ (NE) previous action (either positive or negative). The probability of taking a certain decision is calculated using BN2. Walking to another location to collected water has a lower efficacy compared to boiling the water and this has a lower efficacy compared to buying bottled water. The household agents’ income level determines which decision option is more likely to be taken since the costs of the options differ. River water is free of cost, boiling water has a price tag and so has buying bottled water.

The mathematical formula of BN2 for computing the decision can be expressed as:

|  | $\boldsymbol{P} \left( \boldsymbol{D} \vert\boldsymbol{I, E, OE, NE} \right)\boldsymbol{=}\frac{\boldsymbol{P} \left( \boldsymbol{I, E, OE, NE} \vert\boldsymbol{D} \right)\boldsymbol{P} \boldsymbol{(D)}}{\boldsymbol{P(I, E, OE, NE)}}$ | (3) |
| --- | --- | --- |

### Sensing

Agents sense the visual pollution at the water collection point to judge whether or not the water looks visually polluted. It is not possible to see if water is infected with cholera. However, both data gathered from the online surveys and agents in the CABM confirmed that visual observations of the spatial environment affect the perception and decisions of people as well as agents [6].

Via the media, they sense the general level of infection in the complete area. Agents use communication with their neighbors as a proxy for the number of infections in their own neighborhood.

### Adaptation

In this model, agents adapt their choice of water based on the risk they perceive and their income level. Four sources of water are implemented: river water (different locations), boiled water, tap water and bottled water. In this model, the information used in the threat appraisal and coping appraisal stage of PMT can come from three different sources: Prior Knowledge (1), Communication with others (2) and from the Spatial environment (3).

*Prior knowledge* is based on memory and education level (educated/uneducated). It is used in both the threat appraisal and coping appraisal stages. Persons can recall similar situations from their past and will recall the risk perception they experienced during this incident. They also use memory during the coping appraisal stage, when their last decision was effective, they are likely to repeat it. The education level of a person is a source of prior knowledge of a person. It has a direct influence on the process of making decisions. It affects the way people cope with the perceived risk [7].

*Communication* with others includes: personal communication with neighbors and media. Communication is used in the threat appraisal because it will inform the agent about a certain problem and the severity of this problem. Again, it can also be used during the coping appraisal as the person may receive information via the media on how to reduce risk (in this case the risk of infection). The media agent is activated from day 22 onwards (based on empirical data) and household agents will receive this information.

The last category of information is the spatial environment. In this research, agents visually detect the pollution level of the water (Visual Pollution). This is part of the threat appraisal part. Spatial knowledge can also be useful during the coping appraisal. When a person has a certain level of understanding of the landscape, this person might walk upstream to get to a spring with cleaner water.

Adaptation is implemented via two Bayesian Networks which are further in the section “Learning”.

Household agents perceive the risk of getting infected daily. When agents collect water, they update their internal model with information from neighbors and media and estimate the pollution around the water collection point.

### Interaction

Social interactions have a significant effect on behavior. Household agents might exchange news about the spread of cholera, what preventive decisions they made and their own experience with cholera. Personal communication of agents via their social interaction is divided into two groups:

1. Communication with their households’ members: agents are aware of whether there are sick individuals in their households or not.
2. Communication with their direct neighbours: agents are assigned neighbours who reside in the same community and share the same water collection point.

No data is available on the number of daily contacts of Kumasi resident. However, in a recent study of Melegaro et al. [8], they conducted a survey of daily contacts in Manicaland, Zimbabwe and reported 10.8 contacts per person/day including the contacts with household members. If we consider this rate for our study, excluding the number of household members (average of 3.9), then approximately 7 contacts with neighbors per day will be applied for our cholera model. These seven neighbors are chosen randomly every day. Household agents are assumed to have total awareness of the cholera cases happening within the neighbors’ subset from contacts they make. This interaction will enable agents to perceive the infection level of the water their neighbors used and let them safe or infected. In addition, households’ contacts help agents to gain information on adaptation decisions their neighbors made and how effective is their decision in protecting them.

### Collectives

Groups can be defined in different ways and at different hierarchical levels. In this model we have four levels of organization, the **individual agent**, the **household (a group of individuals)**, **groups** (consisting of several households) and **communities** that consist of a number of groups.

Households consist of some individuals that share the same water source and home location. Households are created at initialization. Individuals are assigned to the households, starting with an adult female as the head of the household followed by randomly adding additional members. Individuals inherit the household features besides those of themselves (S1 Table 1). The distribution of household size is based on information from the Ghana Statistical Service (2012) [9]. These households are distributed over the communities using empirical data on income levels and assigned a building.

In CABM **household** agents living in the same community are grouped based on their income and education level since coping behavior depends on these factors. All neighbors that share the same water fetching point and are living in the same community are assigned to the same group in CABM. Communities are based on empirical data and correspond to the administrative areas of Kumasi city. The study area has 21 communities. A single community can have several groups, created based on agent characteristics and distance to water fetching points.

Household agents may learn individually or collectively with or without interacting with other household agents. To learning collectively, household agents formulate groups. The groups can be in two forms: decentralized (decision making is based on a ‘majority vote’), or centralized in which one agent (leader) uses ML to learn for the whole group to accomplish the group task. Both individuals and groups may learn by either taking information from their social networks (interactive) - i.e. have it as an additional source of information in their ML algorithms - or not (isolated).

### Emergence

Patterns that should emerge are an epidemic curve with a certain shape; risk perception curve of a certain shape; the spatial pattern of percentage of cases in certain locations and the duration of infection; and model performance.

**Disease Diffusion**

The epidemic curves, duration of infection, total cases, and peak days are the most common measures of disease diffusion. An *Epidemic curve* is a graph representation of the distribution of infected cases over an epidemic period [10]. It is a useful way to assign the type of epidemic, calculate the difference between the minimum and maximum incubation period and determine the possible time of exposure. We validated the simulated epidemic curves from all eight models against the empirical epidemic curve.

**Risk Perception**

There is less empirical data about risk perception of infectious diseases compared to other risks, such as environmental risks [11]. Data on disease risk perception is rarely collected in the early stages of epidemics, especially in developing countries [12].

Due to lack of empirical data, two proxy datasets were used, collected via surveys. To construct and train our ML algorithms – Bayesian networks here – we used data from two MOOC surveys [5]. Reported responses of survey participants about the factors (or combination of factors) influencing their risk perception are used to drive the assessment of disease risks (BN1) of household agents. Using the simulated data from eight models, we measure risk perception as the percentage of agents who perceived risk on a given day as high and plot this as a risk perception curve. BN1 evaluates perceived disease risk and returns a value between 0-1; we consider risk perceptions above 0.5 as high.

We assume that the peaks of the risk perception curve and the epidemic curves should be correlated. We based the phases of the risk perception curve on the model of [13,14] as seen in S1 Fig 5.


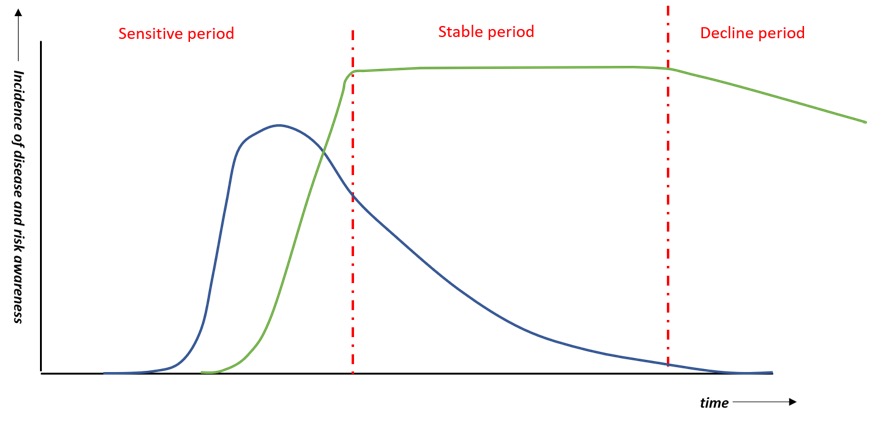


**S1 Fig 5: The proposed model of describing the relation between Epidemic curve and Rik perception curve; the blue line is the epidemic curve and the green line is the associated risk perception curve**

The following assumptions were made about the correlation between the risk perception curve and the epidemic curve:

- Risk perception must not start before the increase in disease incidence.
- Risk perception curve will follow the epidemic curve with a certain amount of time (time delay).
- Risk perception curve will flatten out after the epidemic curve has reached its peak.
- Forgotten period will occur after the end of simulation.

**Spatial Patterns**

We assess the accuracy of our eight models using $\mathrm{SpI}$. It calculates the spatial distribution of infected cases in both real dataset and the outcomes of the simulations [1] to understand how well each type of learning (M1 – M8) complies with the real number of cholera cases per community. The cases were confirmed by bacteriological tests and were registered by the Disease Control Unit (DCU) in Kumasi Ghana during the 2005 epidemic. A simulation with $\mathrm{SpI}$= 1 indicates a perfect reproduction of the actual epidemic (While we do have real data on recorded cholera cases in Kumasi’s 2005 epidemic, the data quality is far from perfect. It is likely to under-represent the extent of the epidemic. Still, this is the best of what exists in the context of a developing country). $\mathrm{SpI}$ is calculated using equation 4:

|  | $\mathrm{SpI}= \frac{\sum_{i=0}^{n} (\left( {CC}_{s,i}- \overline{{CC}_{s}} \right)\left( {CC}_{d,i}- \overline{{CC}_{d}} \right))}{\sqrt{\sum_{i=1}^{n} {({CC}_{s,i}-\overline{{CC}_{s}})}^{2}\sum_{i=1}^{n} {({CC}_{d,i}- \overline{{CC}_{d}})}^{2}}}$ | *(4)* |
| --- | --- | --- |

Where $CC$ is the relative percentage of diagnosed disease cases per community, $s$ refers to the simulation scenario and $d$ to the real diagnosed cases in 2005. The underscore refers to the average values. The index $i$ refers to a community $i$ and $n$ is the total number of infected across all communities. In addition, we will show maps, in which we present the spatial distribution of the coping appraisal decisions type per community per model.

### Stochasticity

This model contains different sources of stochasticity both in the generation of the synthetic population and in the model itself. Within the model there are two sources of stochasticity that require mentioning: the contacts of agents with neighbors are randomly generated and the time of fetching water (in relation to rain) is selected randomly every day. Every day, the time to fetch water is changed to a random value between (6 am to 10 pm), and it should be after the rain stopped. Also, the time of rain differs from day to day. It can be any random time between 0 – 23 hours. All individual agents are assumed to use the water collected by the household and at the start of the simulation all individuals are susceptible to cholera.

S1 Fig 6 shows the stability of running CABM 100 times. We evaluated how the average number of infected cases on the epidemic peak day changes by increasing the number of random seeds run.


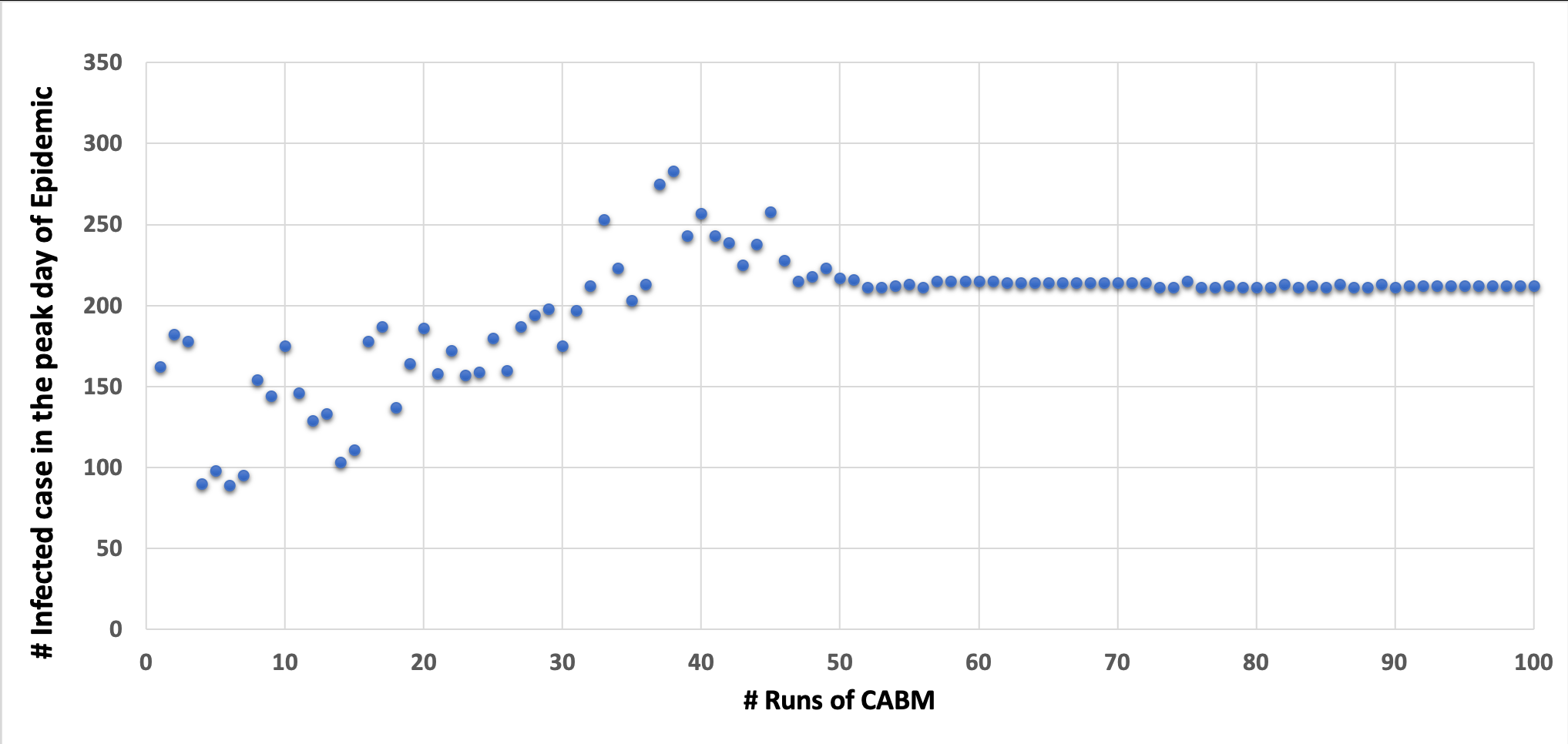


**S1 Fig 6: Stability check of running CABM 100 times**

The dots represent the average across the runs (S1 Fig 6). The value of the epidemic peak stabilizes after about 53 runs. This confirms that the results averaged across 100 runs are very stable.

### Observation

To evaluate the difference between group-learning vs individual-learning and interactive-learning vs isolated-learning, we record for each run which household belongs to which group and if it concerned isolated or interactive learning. The following observations can be retrieved from the sub-models (described further under sub-models):

Disease spread:

1. The number of infections per time step (including the spatial distribution of the infection).
2. From the above information we can derive the epidemic curve for each run, the percentage of infection per community, the duration of infection per community and the day of infection per community.

Visual pollution model:

1. The visual pollution of the water collection points during each time step.

Hydrological model:

1. The infection level of each water collection point during each time step.
2. The infection level of each open dumpsite during every time step.
3. Total discharge over time.

By comparing the output of the visual pollution model with the actual infection level, we can determine the effectiveness of the visual observations for disease prevention.

Activity model:

1. The number of agents who perceive risk per time step, including their spatial and economic distribution.
2. The choice of water source of each household per times step and in case of river water, the water collection point where this water was retrieved from the river.

## Details

### Implementation details

The CABM is implemented in NetLogo (version 5.2.0) and the learning algorithm (both BN1 and BN2) are coded using R statistical language. During each model run, household agents in NetLogo collect their BNs’ inputs (e.g. estimate pollution levels, retrieve own memory, check own health status, etc.), and call their BNs via the R extension of NetLogo. After processing the BNs, R returns the information on risk perception and disease coping strategies back to the agents in NetLogo.

### Initialization

During initialization, households (default is 8500 that represents 12.7% of the actual number) with a corresponding number of individuals are created (about 33,800 individuals). Individuals are assigned to the households, starting with an adult female as the head of the household followed by randomly adding additional members. Individuals inherit the household features besides those of themselves (S1 Table 1). The distribution of household size is based on information from the Ghana Statistical Service [58]. These households are distributed over the communities using empirical data on income levels and assigned a building. The hygiene levels (low, medium, and high) of household agents are derived during the calibration stage [1]. The hygiene levels are distributed as 19% (low), 52% (medium), and 29% (high). After the creation of a synthetic population and the distribution of the agents over the study area, households calculate the nearest water collection point and the nearest dumpsite.

The census data of Kumasi, Ghana includes the income distribution. The distribution of the three levels is as 19% (low), 52% (medium), and 29% (high). However, we exclude high-level incomes since they will not use river water. Therefore, by scaling both medium and low-income levels we will get 73% and 27% respectively (which represent 71% of total simulated households). In addition, 14 % of low- and middle-income level households do not have access to tap water. The following table (S1 Table 3) shows the parameters of this cholera model:

S1 Table **3**: CABM parameters (Abdulkareem et al., 2018)

| *New Parameters* | *Value* | *Description* |
| --- | --- | --- |
| *Literacy rate* | 74.1 % | (Ghana Statistical Service 2012) |
| *Media* | Activation on day 22 | During the 2005 outbreak, newspapers and TV channels published news about the cholera in the region after about three weeks of epidemic started (visit: [Ghana News Archive](https://www.modernghana.com/news/88253/1/cholera-outbreak-in-accra-.html)). |
| *Waste collection* | 85% of dumpsites | 85% of waste is collected by Kumasi municipality [15]. The rest remains uncollected for a week or more. |
| *Amount of garbage* | 2.925 Kg/household/day | derived from literature [16]. |
| *Number of contacts with neighbors* | 7 neighbors | Derived from literature [8]. |

At initialization all spatial environments will be loaded, including rainfall data (S1 Fig 7). The infection level of the dumpsites will be set to zero. All river water is assumed to be clean.


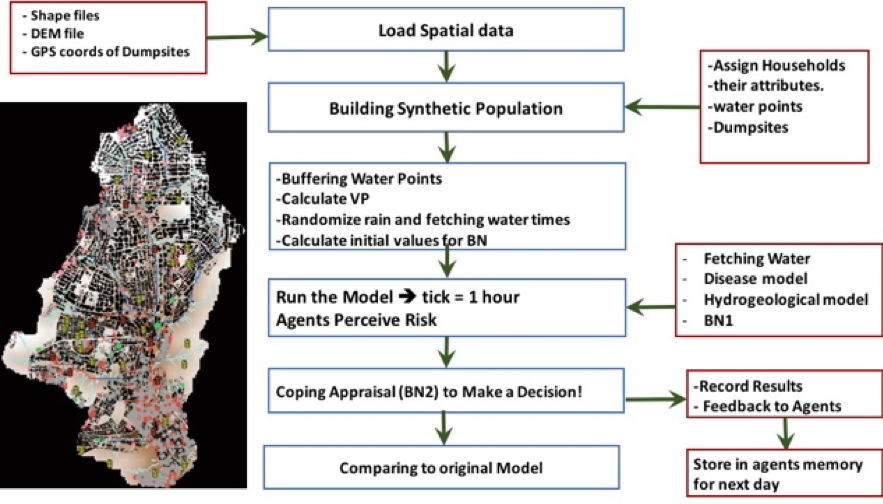


**S1 Fig 7: CABM setup and running flow adjusted from [17]**

### Input data

For the CABM, data from the Ghana Bureau of Statistics [58] is used to create the synthetic population of individuals and households. Data on access to tap water was derived from statistical information of the Ghana Statistical Service [58]. The dataset of confirmed Cholera cases for the 2005 epidemics were confirmed by a bacteriological test and were reported to the Disease Control Unit (DCU) by reporting facilities [18]. The DEM was downloaded from [CGIAR website](http://srtm.csi.cgiar.org/) as a Geotiff image. Flow direction and flow accumulation layers have been calculated based on this DEM using ArcGIS. Houses were digitized based on the Google image of the area of the year 2006, and refuse dump locations have been collected using GPS [19].

The data of rainfall was gained from Tutiempo Network SL, that provided registered data from 30^th^ of September to 30^th^ of November, 2005. In Augustijn et al (2016), an assumption was made of two hours of rainfall per day since no information was available regarding hourly rainfall.

#### Learning data

Perceiving risk has a great impact on the spread of diseases [20]. However, data on how people behave and perceive risk during epidemics are not available in most cases. There is a demand for micro-level behavioral datasets that describe the relationship between risk perception and model variables. To obtain the data that was necessary to construct the structure of the BNs and train the learning algorithm we conducted two surveys: a survey among international participants of the Massive Open Online Course (MOOC) Geohealth and an online survey (Google survey). MOOC survey was run two times: in 2016 and 2017 with 194 and 235 participants from 92 countries respectively, while the Google survey was run in 2017 with 125 participants from 33 countries [5].

In the MOOC survey, four levels of pollution were shown through pictures: no visual pollution, brown water, low visual pollution and high visual pollution. While, in the Google survey, participants only knew that the water was polluted, without any indication of the level of pollution. Thus, we combined the data gathered from these two surveys into one dataset to ensure that all possible combinations of factors were stated and risk perception responses were included.

The participants of both surveys are well-educated people. However, results show that participants evaluated the quality of the water by its appearance. This confirms the assumption that low income population of Kumasi does the same. We use our abstract eyes to judge the surrounding and make conclusions in certain situations.

The datasets gathered from the surveys were used to train and validate the learning algorithm (in our case the Bayesian Networks) to be integrated with CABM to steer the behavior of household agents during the simulation. In addition, they were also used to validate the risk perception and coping appraisal of household agents in the simulation [5].

### Sub-models

#### Disease Spread Model

The cholera infection spreads via infected water in two ways either Environment – to – Human (EH) or Human – to – Environment – to – Human (HEH) that corresponds to a hyper infectious state and normal infectious state. By default, the probability of fetching EH polluted water with cholera is 3 % (*the value for the probability of fetching EH contaminated water was determined by running the model with a range of input values (1– 6%) to identify the minimum value for which the output was stable, which was 3%.*). To illustrate the selection of the value for the probability of fetching EH polluted water we refer to the S1 Fig 8 that was tested in [1]. However, the probability of fetching HEH infected water relies on the runoff from infected dumpsites that is varying in space and time. It is assumed that the infection level of a dumpsite is increased after dumping infected waste. This will lead dumpsites to induce runoff particles with hyper infectious bacteria if the infection level of the dumpsite is above the threshold.


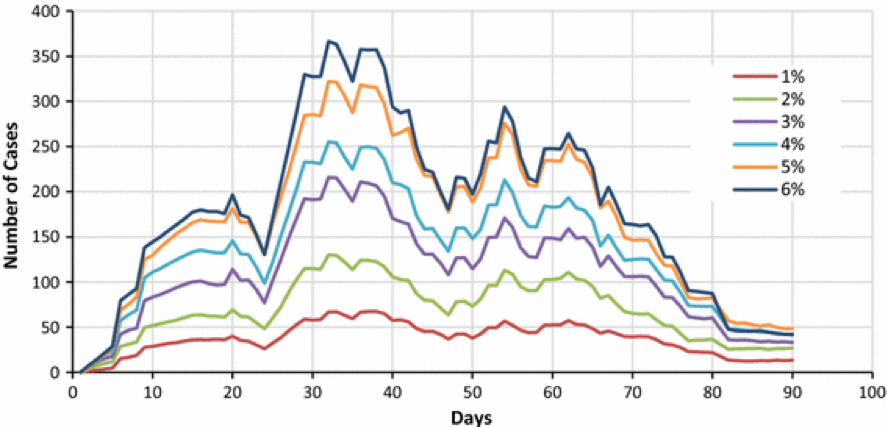


**S1 Fig 8: Identifying the minimum value to stabilize the CABM with a range of input values (1 – 6%) [1]**

All individuals in the model are considered to be susceptible at the beginning of the simulation. Therefore, the infection may occur when they use/drink contaminated water that has been fetched from the river. The sub-model of cholera spread in CABM have been described in more details in [1]. The disease model is based on adjusted SEIR model as it had been described in section 2.1

#### Visual Pollution Model

Visual pollution of river water at the water collection points is used as a proxy for households to judge the safety of the river water. The visual pollution level is calculated based on the combined link order of the river and the number of open refuse dumps that are located within a distance of 200 meters from the river. It is common in Kumasi to observe waste dumps located on the river banks or in the river path [21].  VP is calculated based on the following equation:

|  | $\mathbf{f}\left( \mathbf{VP} \right)\mathbf{=}\sum_{\mathbf{i=1}}^{\mathbf{N}} \frac{\mathbf{x}_{\boldsymbol{i}} \mathbf{g}_{\boldsymbol{i}}}{\boldsymbol{d}_{\boldsymbol{i}}}$ | (5) |
| --- | --- | --- |

Where *N* is the number of dumpsites around the water collection points; *x* is the number of households who use the dumpsite; *g* is the amount of garbage produced by each household; and *d* is the distance from the dumpsites to the water point (1 m ≤ $d$ ≤ 200 m).

The number of dumpsites is fixed throughout the simulation and the number of households will also remain the same over a simulation run. During dry days, we assume that the amount of garbage across the river remains static. However, the visual pollution level is dynamic. This dynamic is due to the random selection of dumpsites that will be cleared. Only 85% of waste is collected twice a week in Kumasi [15]. In addition, during heavy rainfall days, the river will carry floating garbage to downstream areas. This also leads to a changeability in visual pollution levels at all water collection points over time. This sub-model is described in more detail in [6] and in chapter four in [22].

#### Hydrological Model

This model simulates the flow of hyper-infectious bacteria from dumpsites towards the downstream [1]. Therefore, several environmental layers, including a DEM, flow direction layer, and flow accumulation layer, are loaded into the model during initialization. The DEM layer is used to direct the movement of rain particles downstream. The data of actual rainfall of the study area provides the model with the real amount of surface runoff, and the volume of water per rain particle agent is computed by dividing the total volume of rain by the number of rain particle agents. The flow of a rain particle agent to a neighboring cell is determined by the flow direction layer using the steepest slope. While the travel time of rain particle agents is calculated using the general Manning formulas: sheet flow, gully flow, and river flow that reflects different travel time.

#### Activity Model

In CABM, we assumed that household agents with a high-income level are safe since they buy bottled water when cholera starts to spread. However, household agents with a medium or low-income that have no access to tap water use intelligent learning to perceive risk (BN1) and to choose a coping strategy (BN2). Household agents gain information from the spatial environment, namely the visual pollution at water collection points, from the media (which starts to broadcast news of cholera on day 22), and their own prior knowledge of any cholera cases. During a simulation, household agents may retrieve information via their randomly assigned daily contacts with zero to seven neighbors [8]. When interactive learning is activated, social interactions among household agents help to share information on cholera cases that occurred in their communities, as well as on the effectiveness of coping decisions. Depending on perceived risk (BN1), a household agent either keeps the same behavior (D1 ‘drink from the same water fetching point’), or switches to a protective behavior (D2 ‘walking to a cleaner water point’, D3 ‘boiling fetched water’ or D4 ‘buying bottled water’).

Individuals in households will use the water and might get the infection. Households with infected individuals will dump their waste on the closest dump sites. This can lead to the spread of cholera to the river and as a consequence infection in other households when they fetch contaminated water.

# References:

1. Augustijn EW, Doldersum T, Useya J, Augustijn D. Agent-based modelling of cholera. Stoch Environ Res Risk Assess. 2016;30: 2079–2095. doi:10.1007/s00477-015-1199-x

2. Abdulkareem SA, Augustijn E-W, Mustafa YT, Filatova T. Intelligent judgements over health risks in a spatial agent-based model. Int J Health Geogr. 2018;17: 8. doi:10.1186/s12942-018-0128-x

3. Maddux JE, Rogers RW. Protection motivation and self-efficacy: A revised theory of fear appeals and attitude change. J Exp Soc Psychol. 1983;19: 469–479. doi:10.1016/0022-1031(83)90023-9

4. Rand W. Machine learning meets agent-based modeling: When not to go to a bar. Proc Agent. 2006.

5. Abdulkareem SA, Mustafa YT, Augustijn E-W, Filatova T. Bayesian networks for spatial learning: a workflow on using limited survey data for intelligent learning in spatial agent-based models. Geoinformatica. 2019;23: 243–268. doi:10.1007/s10707-019-00347-0

6. Abdulkareem SA, Augustijn E-W, Mustafa YT, Filatova T. Integrating Spatial Intelligence for risk perception in an Agent Based Disease Model. GeoComputation. 2017; 1–7.

7. Gregson S, Waddell H, Chandiwana S. School education and HIV control in sub-Saharan Africa: From discord to harmony? J Int Dev. 2001;13: 467–485. doi:10.1002/jid.798

8. Melegaro A, Del Fava E, Poletti P, Merler S, Nyamukapa C, Williams J, et al. Social Contact Structures and Time Use Patterns in the Manicaland Province of Zimbabwe. Nishiura H, editor. PLoS One. 2017;12: e0170459. doi:10.1371/journal.pone.0170459

9. Ghana Statistical Service. 2010 Population and Housing Census. Ghana Stat Serv. 2012; 1–117. doi:10.1371/journal.pone.0104053

10. Wilson EB, Burke MH. The Epidemic Curve. Proc Natl Acad Sci U S A. 1943;29: 43–48. doi:10.1073/pnas.29.1.43

11. De Zwart O, Veldhuijzen IK, Elam G, Aro AR, Abraham T, Bishop GD, et al. Perceived threat, risk perception, and efficacy beliefs related to SARS and other (emerging) infectious diseases: Results of an international survey. Int J Behav Med. 2009;16: 30–40. doi:10.1007/s12529-008-9008-2

12. Liao Y, Xu B, Wang J, Liu X. A new method for assessing the risk of infectious disease outbreak. Sci Rep. 2017;7: 40084. doi:10.1038/srep40084

13. Wei J, Wang F, Zhao D. A risk perception model: simulating public response to news reports in China. undefined. 2012.

14. Moinet A, Barrat A, Satorras RP, Pastor-Satorras R, Barrat A, Effect AB. Effect of risk perception on epidemic spreading in temporal networks. Phys Rev E. 2018;97: 012313. doi:10.1103/PhysRevE.97.012313

15. Asase M, Yanful EK, Mensah M, Stanford J, Amponsah S. Comparison of municipal solid waste management systems in Canada and Ghana: A case study of the cities of London, Ontario, and Kumasi, Ghana. Waste Management. 2009. pp. 2779–2786. doi:10.1016/j.wasman.2009.06.019

16. Miezah K, Obiri-Danso K, Kadar Z, Fei-Baffoe B, Mensah MY. Municipal solid waste characterization and quantification as a measure towards effective waste management in Ghana. Waste Manag. 2015;46: 15–27. doi:10.1016/j.wasman.2015.09.009

17. Doldersum T. The role of water in cholera diffusion Improvements of a cholera diffusion model for Kumasi, Ghana. 2013.

18. Osei FB, Duker AA. Spatial dependency of V. cholera prevalence on open space refuse dumps in Kumasi, Ghana: a spatial statistical modelling. Int J Health Geogr. 2008;7: 62. doi:10.1186/1476-072X-7-62

19. Osei FB, Duker AA, Augustijn EW, Stein A. Spatial dependency of cholera prevalence on potential cholera reservoirs in an urban area, Kumasi, Ghana. Int J Appl Earth Obs Geoinf. 2010;12: 331–339. doi:10.1016/j.jag.2010.04.005

20. Kitchovitch S, Liò P, Li P, Liò P. Risk perception and disease spread on social networks. Procedia Comput Sci. 2010;1: 2345–2354. doi:10.1016/j.procs.2010.04.264

21. Danquah L, Abass K, Nikoi AA. Anthropogenic Pollution of Inland Waters: the Case of the Aboabo River in Kumasi, Ghana. J Sustain Dev. 2011;4: 103–115. doi:10.5539/jsd.v4n6p103

22. Abdulkareem SA. Enhancing agent-based models with artificial intelligence for complex decision making. University of Twente. 2019. doi:10.3990/1.9789036547482
