# Supplementary material for: Risk perception and behavioral change during epidemics: Comparing models of individual and collective learning
Source: PLoS One. 2020 Jan 6;15(1):e0226483. doi: 10.1371/journal.pone.0226483 (PMC6944362; doi:10.1371/journal.pone.0226483)
Supplement: S2 Appendix — (DOCX) [file pone.0226483.s002.docx]

## S2 Appendix – Normality test

Every time we use our cholera ABM (CABM) as a test case for our research objectives, we make sure that the methodology we follow is rigid and sound, including the statistical analysis of the results. CABM’s stability had been tested before being integrated with machine learning algorithm [1]. In addition, when Bayesian networks were used to steer the behavior of household agents in Abdulkareem et al (2018), a sensitivity analysis had been used to test the influence of timing and number of communications on agents behavior [2]. Moreover, when behavioral data have been used to construct and train Bayesian networks, we again tested the stability of the models versions [3].

For this article, the statistical analysis was performed on the output data of the models to show and analyze the distributions of the obtained results. This involved normality test of the total number of infected cases. Two tests of normality (Shapiro–Wilk and Lilliefors) were applied to verify the frequency distribution of the infected cases with all the models (S2 Table 1) [4]. In addition, we show the normal Quantile – Quantile (Q-Q) plots and histograms to show the normality of the eight models’ results (S2 Fig 1) [4]. Q-Q plot shows the empirical quantiles of the models’ results against corresponding quantiles of their normal distribution. The Q-Q plot and the histogram of the total of infected cases of one of model show some deviations from normality, but according to the tests of normality, the data of the total of infected cases do not deviate from a normal distribution.

Based on Shapiro-Wilk test a high value of W refers to how closely data follow normal distribution while with Lilliefors, the approve of normality distribution would be with a small value of the statistics D. Therefore, according to the implemented tests, this data is not significantly different from normality.

S2 Table 1: Normality test of the total infected cholera cases resulted from the eight models

| Model | Shapiro-Wilk test | Lilliefors test |
| --- | --- | --- |
| M1: RP&CA (In-I) | p_value = 0.00091 | p_value = 0.08768 |
|  | Statistic (W) = 0.90976 | Statistic (D) = 0.11531 |
| M2: RP&CA (In-N) | p_value = 0.7989 | p_value = 0.9336 |
|  | Statistic (W) = 0.98856 | Statistic (D) = 0.051247 |
| M3: RP&CA (D-I) | p_value = 0.000092 | p_value = 0.006657 |
|  | Statistic (W) = 0.87717 | Statistic (D) = 0.15103 |
| M4: RP&CA (D-N) | p_value = 0.6657 | p_value = 0.4449 |
|  | Statistic (W) = 0.9826 | Statistic (D) = 0.087319 |
| M5: RP&CA (C-I) | p_value = 0.2061 | p_value = 0.6334 |
|  | Statistic (W) = 0.96659 | Statistic (D) = 0.080911 |
| M6: RP&CA (C-N) | p_value = 0.349 | p_value = 0.1164 |
|  | Statistic (W) = 0.97489 | Statistic (D) = 0.11159 |
| M7: RP(D-N), CA (In-N) | p_value = 0.05841 | p_value = 0.1767 |
|  | Statistic (W) = 0.95636 | Statistic (D) = 0.1046 |
| M8: RP(C-N), CA (In-N) | p_value = 0.599 | p_value = 0.7273 |
|  | Statistic (W) = 0.98139 | Statistic (D) = 0.07226 |

The normality test of the eight models shows that M2 proves more stability and results of running the simulation 100 times are normally distributed as the p_value in both Shapiro-Wilk and Lilliefors tests found (S2 Table 1). The simulations’ output of total infected cases of M2 shows high correlation with their corresponding normal scores. Moreover, the Q-Q plot and the histogram of M2 (S2 Fig 1) also show the resulted data are almost normally distributed with very little deviation. The normal distribution of M8 where household agents are guided by their opinion leaders for only risk perception appears to be a good fit to the simulations’ output (S2 Table 1 and S2 Fig 1). The same is observed for M4, M5 and M6 and also M7 (for all of them p_value is more than 0.05).

While results of running M3 shows that they are not normally distributed (p_values of both tests are less than 0.05). This has been also proved with other the highest standard deviation it recoded in Table 4 in the article (SD = 402). In addition, M1 with isolated individual agents also recorded p_value in Shapiro-Wilk test less than 0.05 (S2 Table 1) and its Q-Q plot shows few runs deviated from their normal distribution (S2 Fig 1).

| 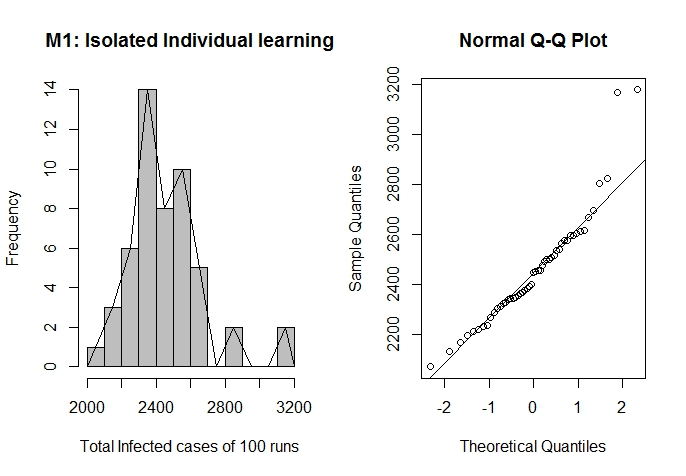 | 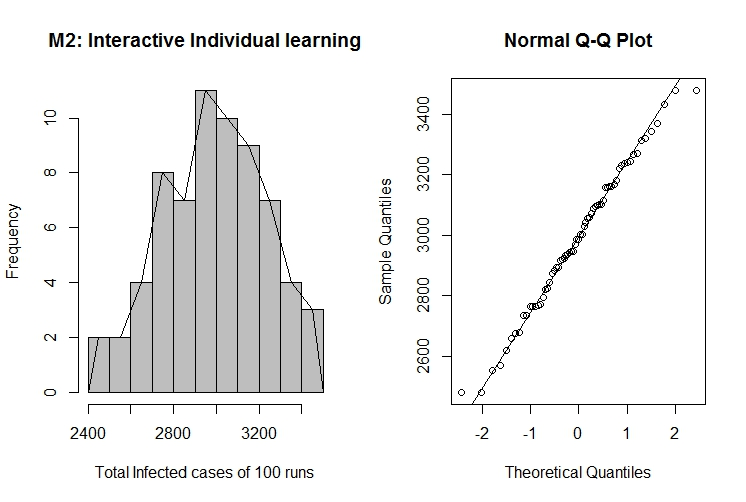 |
| --- | --- |
| 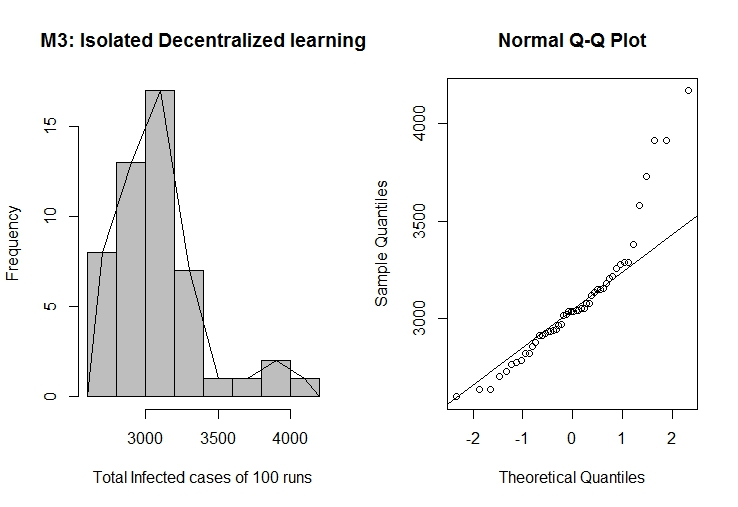 | 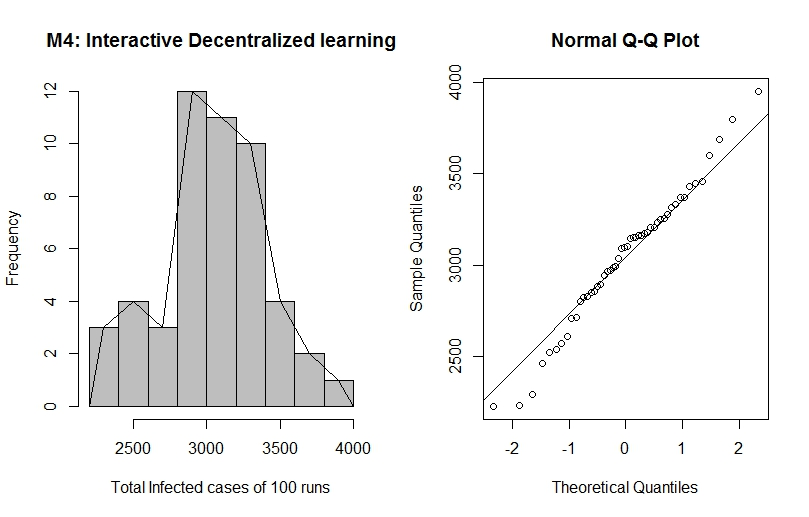 |
| 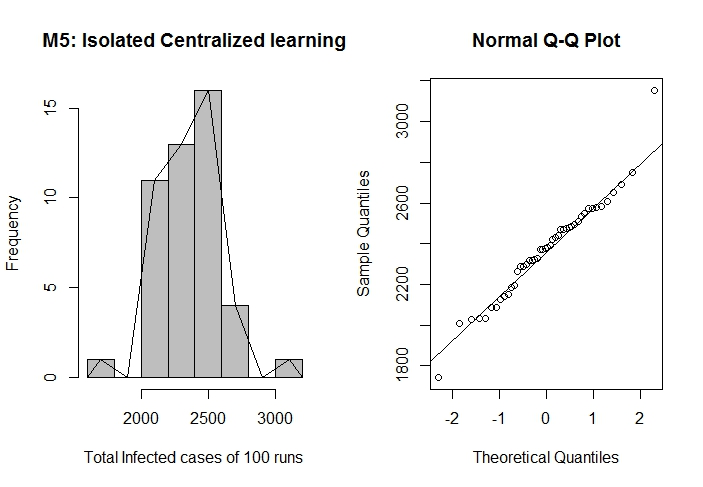 | 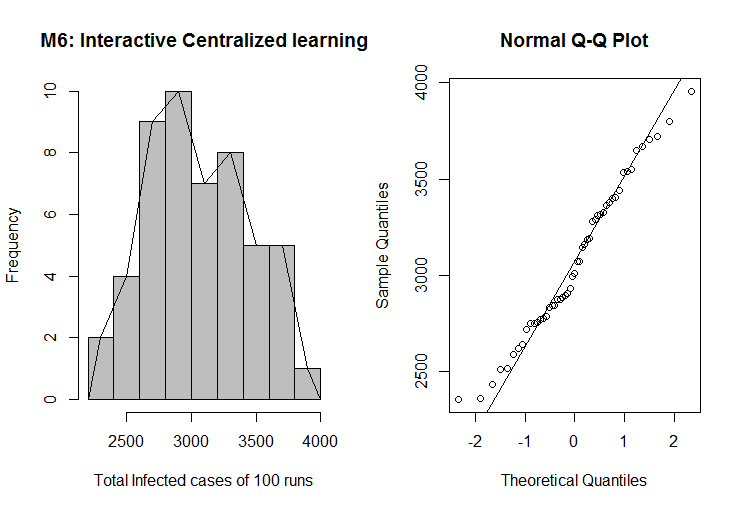 |
| 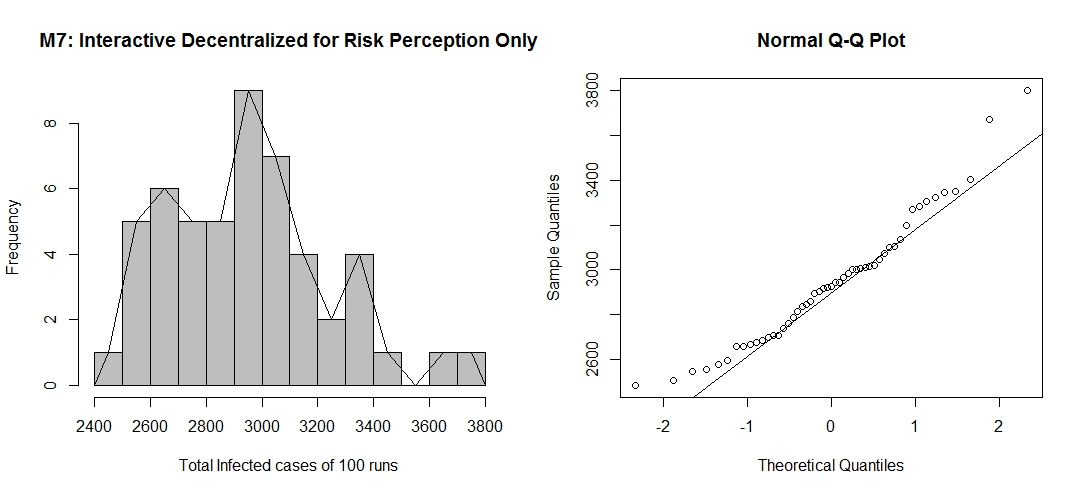 | 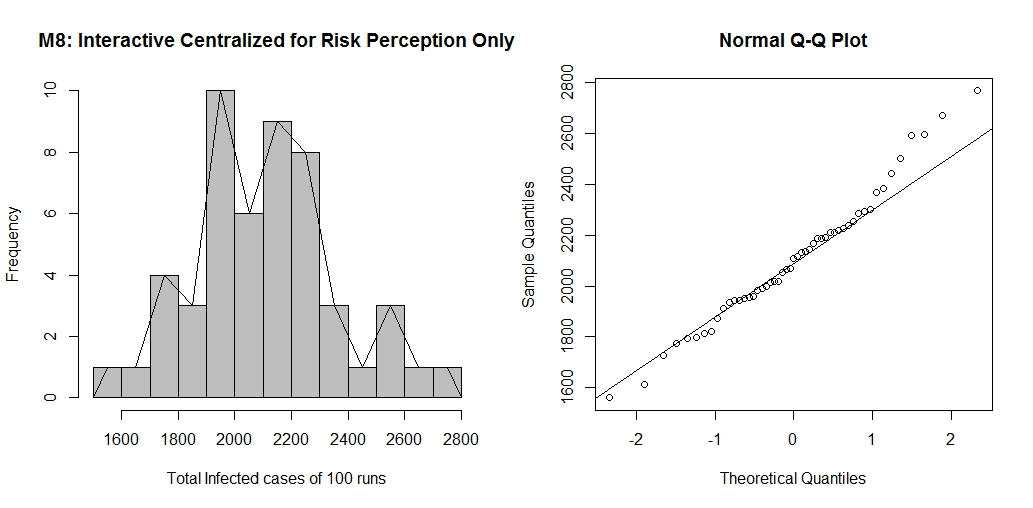 |

**S2 Fig 1: Normal Q - Q plots and histograms with the normal curve of the resulted total infected cases of the eight models (M1 – M8)**

## References

1. Augustijn EW, Doldersum T, Useya J, Augustijn D. Agent-based modelling of cholera. Stoch Environ Res Risk Assess. 2016;30: 2079–2095. doi:10.1007/s00477-015-1199-x

2. Abdulkareem SA, Augustijn E-W, Mustafa YT, Filatova T. Intelligent judgements over health risks in a spatial agent-based model. Int J Health Geogr. 2018;17: 8. doi:10.1186/s12942-018-0128-x

3. Abdulkareem SA, Mustafa YT, Augustijn E-W, Filatova T. Bayesian networks for spatial learning: a workflow on using limited survey data for intelligent learning in spatial agent-based models. Geoinformatica. 2019;23: 243–268. doi:10.1007/s10707-019-00347-0

4. Ghasemi A, Zahediasl S. Normality tests for statistical analysis: A guide for non-statisticians. Int J Endocrinol Metab. 2012;10: 486–489. doi:10.5812/ijem.3505
